# Loss of virulence of *Botrytis cinerea* mutants defective in phytotoxin production is restored by modifying inoculation medium

**DOI:** 10.1128/mbio.03119-25

**Published:** 2025-12-29

**Authors:** Si Qin, Xiaoqian Shi-Kunne, Jie Chen, Frank P. J. Pieterse, Henriek G. Beenen, Yaohua You, Jan A. L. van Kan

**Affiliations:** 1 Laboratory of Phytopathology, Wageningen University 593528 , Wageningen, the Netherlands; 2 Laboratory of Systems and Synthetic Biology, Wageningen University 593528 , Wageningen, the Netherlands; Cornell University, Ithaca, New York, USA

**Keywords:** plant pathogens, transcriptome, virulence factors

## Abstract

Botrydial, botcinic acid, and their derivatives are major phytotoxic metabolites produced by the necrotrophic fungal pathogen *Botrytis cinerea*. These phytotoxins are able to induce programmed cell death in the host and thereby promote plant susceptibility to *B. cinerea*. We observed that a *∆bot2∆boa6* double-mutant strain, which synthesizes neither botrydial nor botcinic acid, was almost avirulent on tomato leaves when the disease assay was performed using synthetic minimal Gamborg B5 medium. However, the virulence of this mutant was restored when the inoculation medium was supplemented with yeast extract. Further virulence assays, which compared the double mutant with other multiple mutants using both inoculation media, revealed a prominent contribution of botrydial and botcinic acid to the full virulence of *B. cinerea*. Therefore, we performed an RNA-sequencing experiment to identify *B. cinerea* genes that contribute to the phenotypic switch from an “incompatible” to a “compatible” interaction between tomato and the *∆bot2∆boa6* double mutant. Four genes encoding cell death-inducing effector proteins were upregulated in *B. cinerea* by the addition of yeast extract, and they were members of a co-expression module that was correlated with the compatible interaction. Functional analyses of these effector genes were performed by overexpressing them individually in the *∆bot2∆boa6* background, followed by disease assays with the Gamborg B5 medium without yeast extract. The overexpression of these four effector genes, individually, did not result in recovery of the expansion of lesions in the absence of yeast extract.

**IMPORTANCE:**

The gray mold fungus *Botrytis cinerea* is a model for necrotrophic plant pathogens due to its wide host range, economic impact, well-assembled genome, and versatile mechanisms for inducing host cell death during colonization. Botrydial and botcinic acid have previously been characterized as major phytotoxins produced by *B. cinerea*. However, studies from different groups reported variable results regarding the contributions of these phytotoxins to fungal virulence. Here, we demonstrate that botrydial and botcinic acid make a prominent contribution to the full virulence of *B. cinerea*, by performing infection assays with mutants that are defective in phytotoxin production using different inoculation media. Supplementation by overexpression of distinct cell death-inducing proteins could not restore the full virulence. This work highlights the pivotal roles of these phytotoxins as compared with other virulence factors, as well as the significant impact of inoculation conditions on compatible and incompatible interactions between the fungus and its hosts.

## INTRODUCTION

The gray mold *Botrytis cinerea* is a plant pathogen that can infect more than 1,000 host species (1). As a necrotroph, *B. cinerea* kills host cells and feeds on dead tissues after penetrating the plant surface. To induce host cell death, *B. cinerea* secretes a cocktail of cell death-inducing molecules (CDIMs), including phytotoxic secondary metabolites (SMs) and cell death-inducing proteins (CDIPs) (2, 3). The most abundantly produced phytotoxic SMs produced by *B. cinerea* are the sesquiterpene botrydial (BOT) and polyketide botcinic acid (BOA) (4–6). CDIPs secreted by *B. cinerea* and their modes of action are more extensively studied than phytotoxic SMs. Thus far, 19 *B. cinerea*-secreted proteins were demonstrated to be capable of inducing cell death in at least one plant species (7–11). However, the number of proteinaceous effectors of *B. cinerea* was predicted to be more than 180 (12), and this fungus may secrete more protein effectors functioning in cell death induction than the currently validated number. Phytotoxic SMs and CDIPs contribute collectively to fungal virulence, with a high functional complementarity (7), but the quantitative contribution of each individual CDIM remains to be clarified.

A study by Dalmais et al. (13) analyzed the roles in virulence of BOT and BOA, either separate or in combination, by testing knockout mutants in key biosynthetic genes. Single mutants defective in the production of either BOT or BOA displayed similar virulence as the wild type (WT), while double mutants that produced neither BOT nor BOA formed ~50% smaller lesions on French bean (13). However, a recent study described no difference in virulence on tomato leaves between a *∆bot2∆boa6* double mutant generated by CRISPR/Cas-mediated transformation and WT (7). The difference between studies suggests that the role of BOT and BOA in virulence of *B. cinerea* requires further investigation. Commonly, the contribution of (single or multiple) genes to fungal virulence is tested by disease assays in which lesion sizes of a WT recipient fungus are compared with mutants lacking the gene(s) encoding putative virulence factors. Such assays can be executed under different conditions, with variations by different laboratories either in the inoculation medium, fungal tissue (mycelial plugs or conidia), plant growth conditions, or the incubation after inoculation. Although these previous studies both performed assays using conidia suspensions to inoculate tomato leaves, there were differences in the inoculation media, spore density, volume of droplets, and in growth conditions for plants (7, 13). We aimed to obtain a better understanding of the molecular basis for the fact that different inoculation conditions with the same set of mutants yield such different results. Here, we describe that inoculation of the *∆bot2∆boa6* double mutant in a synthetic medium can result in restriction of the fungus to necrotic spots at the inoculation site (“incompatible interaction”), whereas supplementation of the medium with yeast extract restored the development of expanding lesions (“compatible interaction”). To obtain insights into the mechanisms that regulate such unexpected (binary) outcome of an inoculation, we performed an RNA-seq study. We inoculated the *∆bot2∆boa6* double mutant on tomato leaves in two distinct inoculation media resulting in either compatible or incompatible interactions. As a control, the WT was inoculated in the same media, which in both cases resulted in a compatible interaction on tomato leaves. Inoculated leaves were sampled during the early interaction phases for RNA extraction and sequencing. Resulting data were analyzed to investigate which differences in transcript profiles may underlie the distinction between compatible and incompatible interactions.

## RESULTS

### The importance of BOT and BOA for fungal virulence depends on the inoculation medium

**Fig 1:**
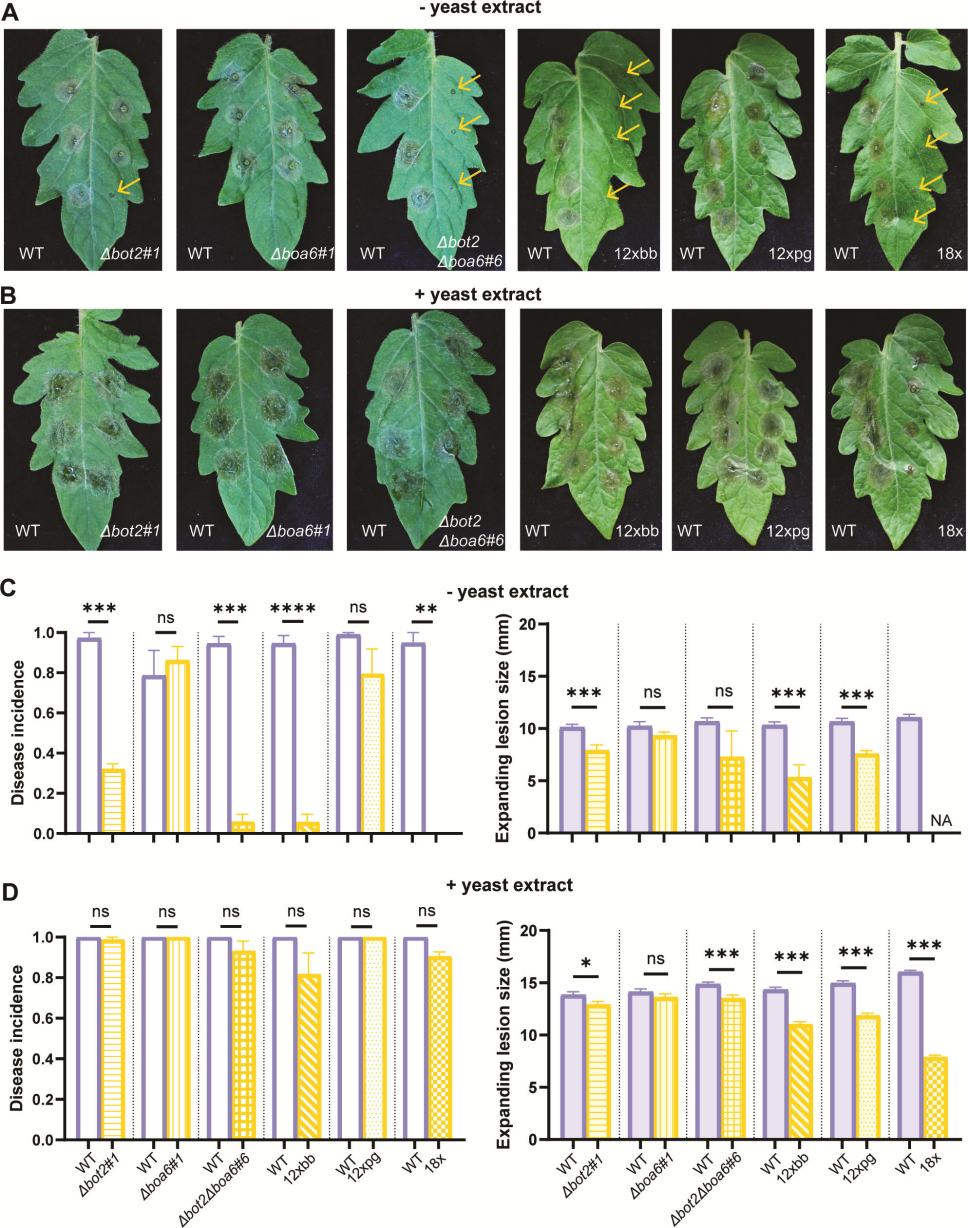
Infection assays to compare the virulence of *∆bot2#1, ∆boa6#1,* and *∆bot2∆boa6#6* and 12xbb, 12xpg, and 18x mutants with WT B05.10 on tomato leaves using two inoculation media. The assays were performed using Gamborg B5 medium without yeast extract (**A, C**) or with 0.1% yeast extract (**B, D**). Symptoms of tomato leaflets infected by each mutant strain (on the right of the central vein) compared with B05.10 (on the left of the central vein) were photographed at 3 days post inoculation (dpi) (**A, B**). Yellow arrows in panel **A** indicate non-expanding lesions. Bar charts of disease incidences (left) and lesion sizes measured by a digital caliper (right) at 3 dpi present the means with standard errors from 72 (for *∆bot2#1, ∆boa6#1,* and *∆bot2∆boa6#6* compared with WT fungus) or 96 inoculations (for 12xbb, 12xpg, and 18x mutants compared to WT fungus) collected from three independent experiments (**C, D**). The virulence assays for *∆bot2#1, ∆boa6#1,* and *∆bot2∆boa6#6* were performed separately from the experiments for 12xbb, 12xpg, and 18x mutants. Disease incidence was calculated as the ratio of the number of expanding lesions to the total number of inoculated spots. Lesions showing a diameter no larger than 2 mm were considered as non-expanding lesions. Statistical analyses were performed by *t*-test, and the results are shown by either asterisks indicating the significant differences (*P < 0.05, **P < 0.01, ***P < 0.001, ****P < 0.0001) or ns indicating no significance.

The BOT gene cluster in *B. cinerea* consists of seven genes, including the gene *Bcbot2*, which encodes a sesquiterpene cyclase that converts the precursor farnesyl diphosphate (FPP) to presilphiperfolan-8β-ol (14, 15). The BOA gene cluster contains 13 genes (16, 17)*,* of which *Bcboa6* and *Bcboa9* encode polyketide synthases that are key enzymes for BOA biosynthesis (13). We performed infection assays by inoculating tomato leaves with the *∆bot2#1* mutant that cannot produce BOT, the *∆boa6#1* mutant that produces no BOA, and the *∆bot2∆boa6#6* double mutant that produces neither BOT nor BOA. The infection assay was performed using a similar medium to previous studies describing *B. cinerea* infection assays (7, 13). More than 80% of the spots inoculated with WT B05.10 produced expanding lesions (Fig. 1A and C). The *∆boa6#1* single knockout mutant displayed similar disease incidence and expanding lesion sizes as WT (Fig. 1A and C), suggesting that BOA alone does not make a detectable contribution to virulence on tomato. By contrast, ~70% of the primary lesions formed by the *∆bot2#1* mutant did not expand beyond the inoculation spot, while ~97% of primary lesions formed by WT did expand. The ~30% expanding lesions that the *∆bot2#1* mutant developed were ~25% smaller in size as compared to WT (Fig. 1A and C). This observation suggested that production of BOT contributes strongly to the ability to expand beyond the inoculation spot, and to some extent to the expansion rate of lesions. Expanding lesions were barely observed on the leaf half inoculated with the *∆bot2∆boa6#6* double mutant (average disease incidence = ~6%) (Fig. 1A and C). Given the small number of quantifiable lesions for the *∆bot2∆boa6#6* mutant and the relatively large variance, the sizes of expanding lesions of the double mutant did not significantly differ from the wild type. These observations indicate that production of BOT or BOA is pivotal for the capacity of *B. cinerea* to cause expanding lesions under these inoculation conditions, with BOT playing a more important role than BOA in virulence.

When the infection assay was performed using the same fungal strains but with addition of 0.1% yeast extract in the inoculation medium, the capacity of *∆bot2#1* and *∆bot2∆boa6#6* mutants to cause expanding lesions was largely restored and comparable to the WT. Under this modified condition (+y), disease incidence of WT increased to 100%, and the lesion size increased by approx. 5 mm compared with the former condition (-y) (Fig. 1B and D). The *∆boa6#1* single knockout mutant displayed similar disease incidence and lesion sizes as WT (Fig. 1B and D). Interestingly, almost all sites inoculated with *∆bot2#1* and *∆bot2∆boa6#6* mutants showed expanding lesions. Lesion sizes of these mutants were slightly but significantly smaller than those of the WT (Fig. 1B and D). As yeast extract is particularly rich in oligopeptides, it was relevant to test whether the effect of yeast extract was caused by the higher availability of amino acids. The supplementation of inoculation medium with a mixture of 20 amino acids restored the disease incidence for the *∆bot2∆boa6#6* mutant to ~80%, but the size of expanding lesions was a little smaller than the WT inoculated in medium with amino acids (Fig. S1A). Similarly, 100% disease incidence was observed for WT, as well as the single and double phytotoxin biosynthetic mutants when inoculation was performed using potato dextrose broth (Fig. S1B), a commonly used medium for fungal infection assays.

The effect of yeast extract on *B. cinerea* germination and appressorium development was tested *in vitro*. The onset of germination and reaching a level of 90% both were accomplished 1 h earlier than in medium without yeast extract (Fig. S2A); however, the proportion of germ tubes that produced appressoria within 6 h was significantly smaller (Fig. S2B). The supplementation of inoculation medium with a mixture of 20 amino acids did not notably alter the germination rate or speed of *B. cinerea* conidia (Fig. S2A), but it significantly reduced the proportion of germ tubes that produced appressoria (Fig. S2B).

### BOT and BOA have a larger impact on the virulence of *B. cinerea* than CDIPs

Two recent studies reported that the contribution of CDIMs of *B. cinerea* displays a high level of functional complementarity, as demonstrated by the phenotypes of 12xbb, 12xpg, and 18x mutants (in which 12, 12, and 18 *B. cinerea* genes are knocked out, respectively). The mutants 12xbb and 18x were also defective in the *Bcbot*2 and *Bcboa*6 genes, while the 12xpg mutant was not (7, 18). Infection assays were performed to compare the virulence of the 12xbb, 12xpg, and 18x mutants to WT under inoculation conditions described above. The *∆bot2∆boa6#6* strain was assessed along with these mutants in the same experiments. When no yeast extract was added to the inoculation medium, the disease incidence for the 12xbb mutant was even lower than for the *∆bot2∆boa6#6* mutant*,* while the 18x mutant was unable to cause even a single expanding lesion on tomato leaves (Fig. 1A and C). The 12xpg mutant caused expanding lesions at ~80% of the inoculation spots, although the lesion sizes were significantly smaller than for the WT (Fig. 1A and C). These observations collectively suggested that the deletion of BOT and BOA caused a major reduction in disease incidence, while the 12 genes missing in the 12xpg played a less pronounced role when tested under this condition (-y). Upon adding 0.1% yeast extract to inoculation medium, disease incidences for *∆bot2∆boa6#6*, 12xbb, and 18x mutants increased to above 80% and were no longer significantly different from WT (Fig. 1B and D); however, there were still notable differences in the sizes of expanding lesions. With this inoculation medium (+y), the reduction in lesion sizes of the 12xpg mutant was similar to the *∆bot2∆boa6#6* mutant but less pronounced than the reduction in lesion sizes of the 18x mutant (Fig. 1B and D). Additional infection assays were performed with yeast extract containing medium to assess the lesion sizes generated by different mutants in pairwise comparisons. In the presence of yeast extract, the quantitative contribution of the phytotoxins BOT and BOA to lesion size development was comparable to that of the 12 CDIP-encoding genes that were knocked out in the 12xpg mutant (Fig. S1B). The 18x mutant showed the most pronounced reduction in lesion size among the tested knockout strains (Fig. 1; Fig. S1B), indicating additive effects on the virulence of *B. cinerea* of the deletion of *Bcssp2, Bccfem1, Bccdi1,* and *Bccrh1* (knocked out in the 18x mutant but not in the other mutants).

### RNA sequencing of compatible and incompatible interactions

Conversion of an incompatible to compatible interaction by addition of yeast extract to the medium highlights the impact of environment on fungal mutant phenotypes. This observation urged us to explore the underlying mechanism(s) in more detail. We hypothesized that the modified inoculation medium altered the transcriptome of the *∆bot2∆boa6#6* mutant, either by suppressing the expression of genes that result in restricting the fungus in a primary lesion or by promoting expression of genes that overrule host resistance leading to fungal restriction. We performed RNA-seq to compare the transcriptome of the *∆bot2∆boa6#6* mutant between the incompatible and compatible interactions. Conidia of the *∆bot2∆boa6#6* mutant and WT B05.10 were suspended in Gamborg B5 medium without or with yeast extract (indicated by “-y” or “+y”, respectively) and inoculated on tomato leaves, which were sampled at 0, 12, 16, and 24 hpi. These time points represent crucial moments in the infection by the WT under these inoculation conditions: at 12 hpi, *B. cinerea* has penetrated the leaf surface and colonizes internal tissue, but host cell death has not yet been initiated; 16 hpi marks the onset of the appearance of necrotic spots on the upper and lower sides of the leaf; 24 hpi marks the onset of lesion expansion beyond the boundaries of inoculation droplets. Mock-inoculated tomato leaves and *in vitro* cultures (both -y and +y media) of *∆bot2∆boa6#6* mutant and WT at the same time points were included as controls. RNA extracted from these samples was used for sequencing (Table S1). A total of 13,754 *B. cinerea* transcripts were analyzed. Principal component analyses (PCA) showed all samples clustered as expected (Fig. S3). Specifically, the biological replicates clustered within time points, and all *in vitro* samples and all *in planta* samples clustered together, with the exception of the *∆bot2∆boa6#6* -y *in planta* sample, which clustered closer to *in vitro* samples. Since we were mainly interested in the yeast extract-induced transition from an incompatible to a compatible interaction with the host, we focused our initial analysis on the *in planta* conditions. To examine the influence of adding yeast extract on the fungal transcriptome, we compared samples that were grown in media -y and +y across all time points, respectively, *in vitro* and *in planta*. The highest number of DEGs was observed at 24 hpi, reflecting that a large number of genes were affected by yeast extract at the late time points, in both WT and mutant samples (Table 1).

**TABLE 1:** Number of differentially expressed genes (DEGs) in different comparisons

Group	*In planta*		*In vitro*	
	Upregulated	Downregulated	Upregulated	Downregulated
WT^*a*^+y^*b*^ (vs) WT-y^*c*^_0h^*d*^	0	0	0	0
WT +y (vs) WT-y_12h^*d*^	85	103	24	7
WT +y (vs) WT-y_16h^*d*^	50	20	7	24
WT +y (vs) WT-y_24h^*d*^	329	455	120	18
△△^*e*^+y (vs) △△-y_0h	3	3	0	0
△△ +y (vs) △△-y_12h	81	186	60	14
△△ +y (vs) △△-y_16h	129	44	70	20
△△ +y (vs) △△-y_24h	771	513	111	77

^*a*^ Wild type.

^*b*^ With yeast extract.

^*c*^ Without yeast extract.

^*d*^ 0, 12, 16, or 24 hpi.

^*e*^ *∆bot2∆boa6#6* double knockout mutant.

**Fig 2:**
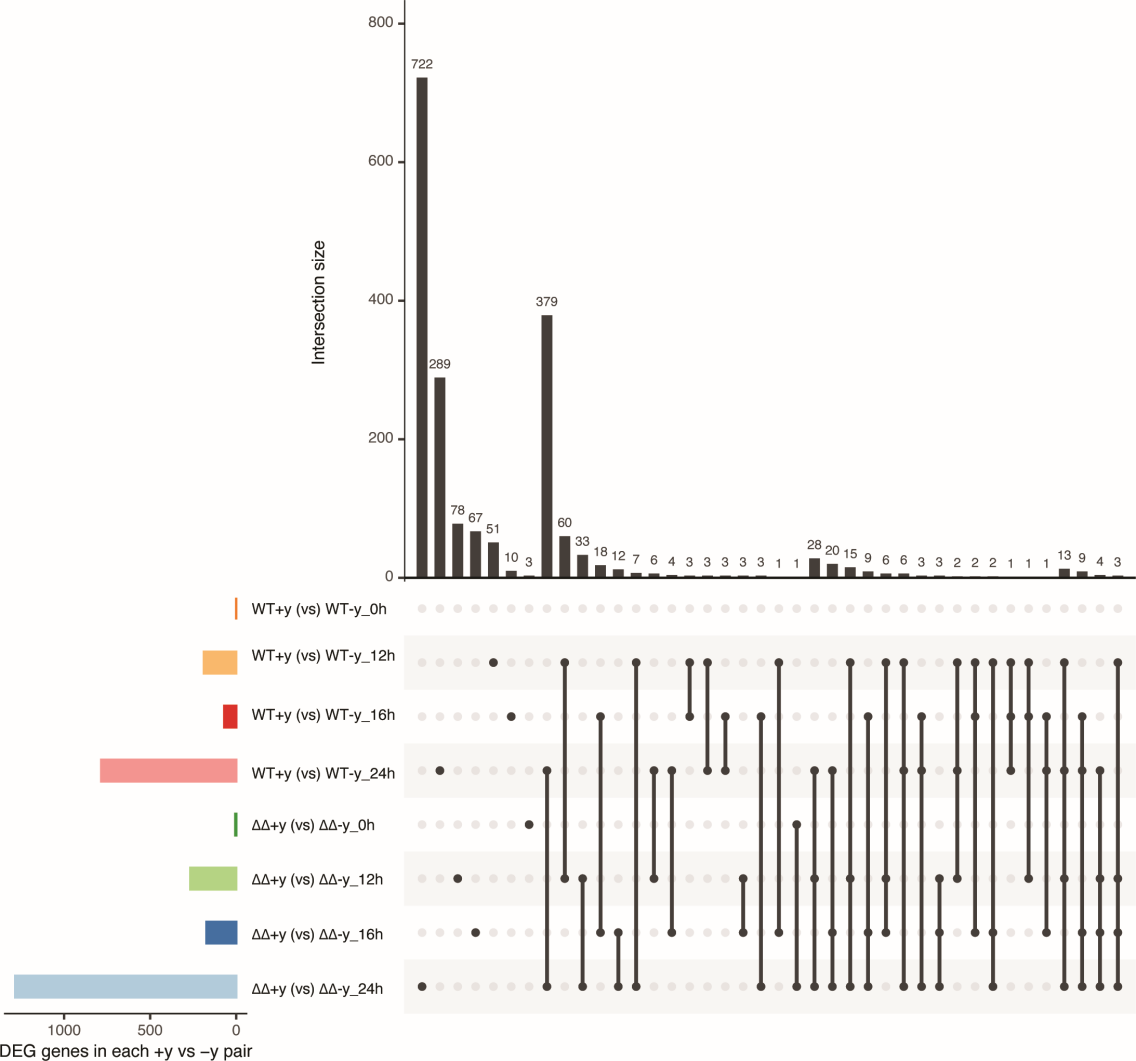
UpSet plot summarizing DEGs of either WT or double mutant between samples with and without yeast at different time points. The vertical bar plot reports the intersection size, and the dot plot reports the set participation in the intersection. Links between different samples show overlapped DEGs between or among compared samples. The bottom left horizontal bar graph shows the total number of DEGs per compared group (set size). Filled dots represent participation in the set.

A large number of the DEGs at 24 hpi (1,491) overlaps between WT and mutant (Fig. 2), which indicates that the fungal strains were similarly affected by the yeast extract. The upregulated genes are mostly enriched in Gene Ontology (GO) terms (*P* < 0.05) related to gene transcription and protein synthesis (Fig. S4). Enriched GO terms detected in the set of down-regulated genes are involved in iron/copper homeostasis. Moreover, *Bcatg13, Bcatg2* (19), and *Bcatg9*, which are involved in autophagy (ATG), were also downregulated. ATG includes a set of programmed cell developmental changes that occur during cellular remodeling and serves as an adaptive response during nutrient starvation (20). In stress conditions, ATG may activate a programmed cell death cascade. The upregulation of transcripts of ATG genes in the mutant upon inoculation without yeast extract at 24 hpi coincides with the restriction of lesion outgrowth. There are also 722 and 289 DEGs that were exclusively differentially expressed in the mutant or WT fungus at 24 hpi, respectively. Enriched GO terms were found only in uniquely downregulated WT and uniquely upregulated mutant gene lists. The enriched GO terms of the genes that were uniquely upregulated in the mutant are related to rRNA processing, protein translation, gene expression, oxidation-reduction, and transmembrane transport (Fig. S5).

### Co-expression network analysis reveals gene clusters correlated with compatible interaction

In order to determine which genes showed similar expression patterns and are co-regulated across all conditions, a co-expression network was created using weighted correlation network analysis (WGCNA). A total of 25 modules of co-expressed genes was obtained, of which the “Gray” module was the residual module, containing all genes that did not show significant correlation in expression profile with genes in other modules (Fig. S6). Evaluation of interconnections between all 25 modules revealed that each module correlated with its own the best, as to be expected (Fig. S7).

**Fig 3:**
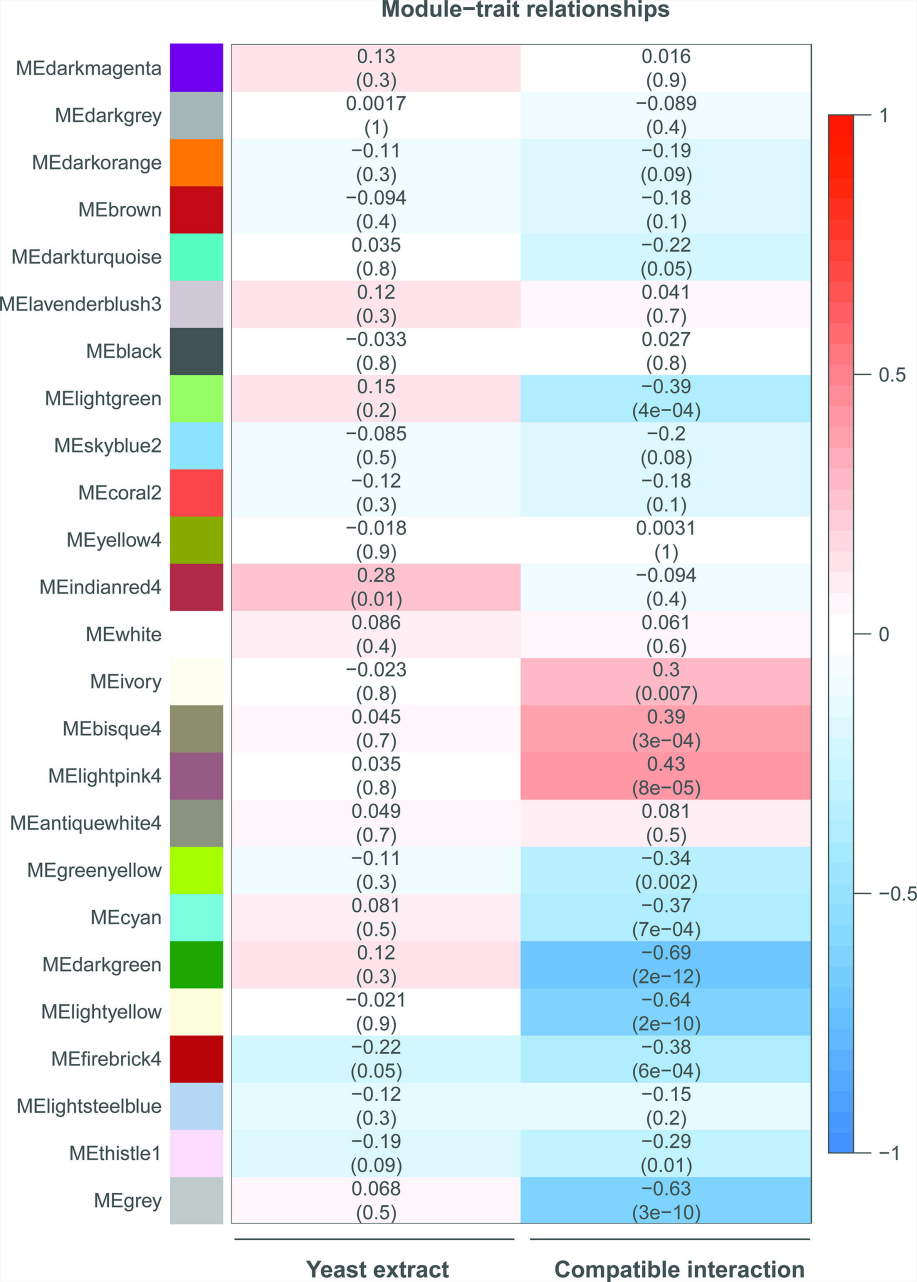
Heatmap showing the correlations of co-expression modules with the presence of yeast extract in the inoculation medium, or in the outcome of the interaction between *B. cinerea* and tomato. On the y-axis are the co-expression modules, with both the names and colors assigned by the WGCNA algorithm. In the heatmap, Pearson correlations with the experimental variables (the presence of yeast extract and outcome of the interaction) are shown, respectively, left and right. Dark red colors indicate a high positive correlation, and dark blue colors indicate a high negative correlation with a condition. Lighter values indicate lower (positive or negative) correlation. For each module, the top line provides the correlation coefficient r, while the lower line (in a bracket) provides the *P*-value for significance.

To analyze which modules might be functionally involved either in the response to the presence of yeast extract in the inoculation medium or in the outcome of the interaction between *B. cinerea* and tomato, we correlated genes of each module with their experimental variables (Fig. 3). There was no significant correlation between any module and the presence of yeast extract (left panel). By contrast, nine modules in the right panel showed significant (positive or negative) correlations with the outcome of the infection, i.e., either with a compatible interaction or an incompatible interaction, respectively.

**Fig 4:**
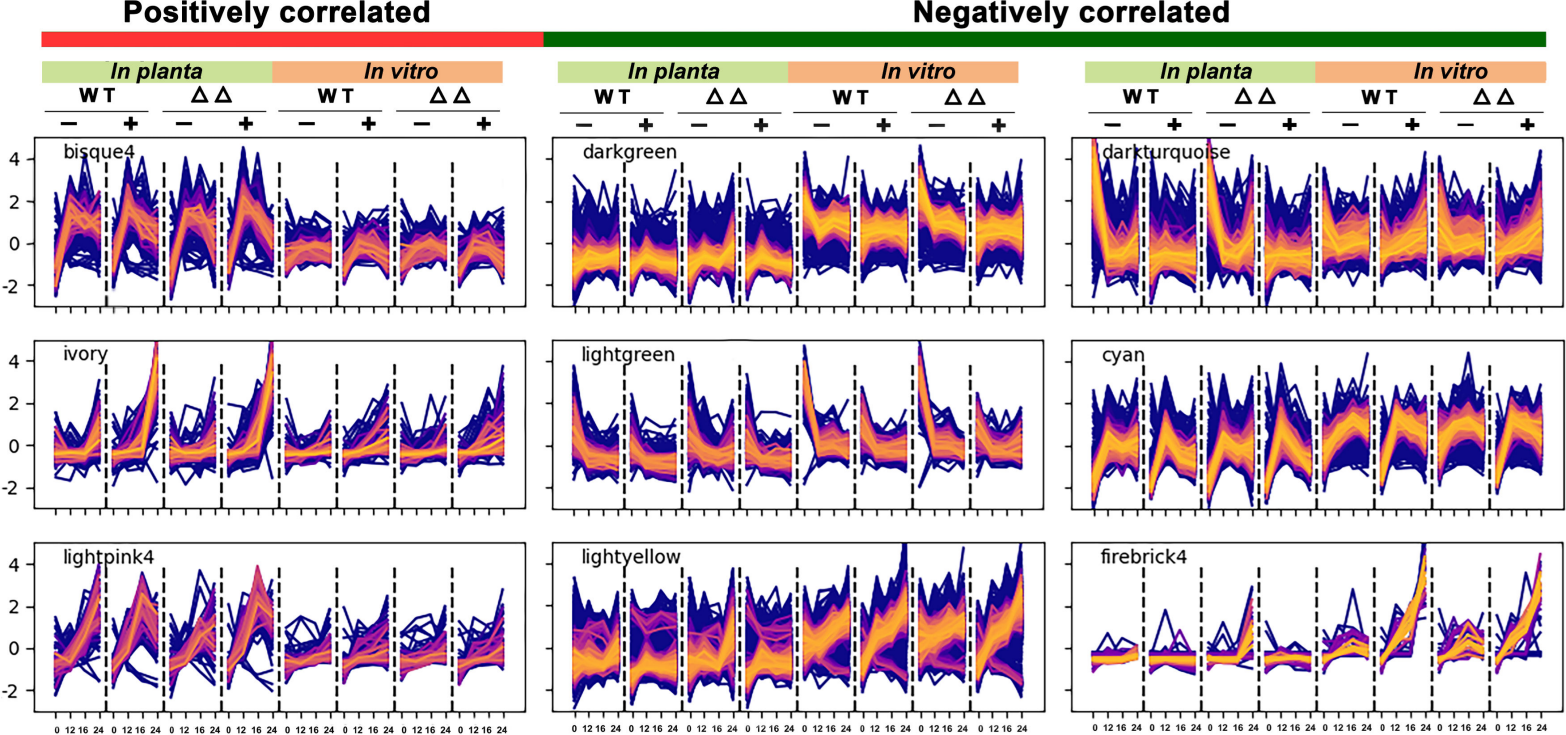
Expression profiles of co-expression modules that show significant (positive or negative) correlations to the compatible interaction between *B. cinerea* and tomato. Each sub-plot contains the full profile of all genes in a module, across all eight conditions. From left to right on the x-axis, the conditions *in planta* and *in vitro* (with the time points 0, 12, 16, and 24 hpi) are WT without yeast extract, WT with yeast extract, double mutant without yeast extract, and double mutant with yeast extract. On the y-axis, the standardized gene expression (z-score) is shown. Each profile is colored according to the assigned module membership value, defined as the correlation with the first principal component of the module profiles (module Eigengene). Lighter colors indicate greater module membership values.

Three modules were positively correlated to the compatible interaction (i.e., conditions in which the lesions expanded), and their expression peaks were more pronounced *in planta* than *in vitro* (Fig. 4). The “bisque4” and “lightpink4” modules contain genes with transient peaks in transcript levels at 12 and 16 hpi, respectively, whereas genes in the “ivory” cluster showed steady transcript levels at 12 and 16 hpi, followed by a strong increase at 24 hpi, especially in the compatible interactions. Interestingly, all three modules are enriched in proteins with a signal peptide for secretion (*P* < 0.01). It should be noted that two modules include genes encoding known CDIPs. Module “bisque4” contains the *Bcnep1, Bcxyg1,* and *Bcplp1* genes, while “ivory” contains the genes *Bcnep2, Bcxyn11A, Bcpg1,* and *Bcssp2* (Data S1). Surprisingly, the module “lightyellow” that showed negative correlation to the compatible interaction contains even more of such CDIP-encoding genes, specifically *Bcspl1, Bchip1, Bcxyl1, Bcgs1, Bcbot2,* and *Bccrh1*.

We examined in detail the three modules positively correlated with the compatible interaction, focusing on their expression profiles, gene content, and GO enrichment. Despite each showing a positive correlation to the compatible interaction, these three modules were quite distinct in their enriched GO terms (Fig. S8). The “bisque4” module contains 245 genes that showed a transient peak in expression *in planta* at 12 hpi, followed by a slight decline, while *in vitro* transcript levels remained fairly constant (Fig. 4). “Bisque4” is enriched in genes involved in mitochondrial activity and splicing. The “lightpink4” module contains 85 genes that showed a transient peak in expression *in planta* at 16 hpi for the +y samples, while transcript levels increased in the -y samples (Fig. 4). The “Lightpink4” has no enriched GO terms, but the gene list contains CAZyme-encoding genes, including pectin methylesterase genes, as well as cytochrome P450-encoding genes, including several from the BOA gene cluster. Genes in the "ivory” cluster (118 genes) showed a continuous increase in transcript levels over infection time points, with the increase more pronounced in the +y samples as compared with -y samples. GO terms enriched in the “ivory” cluster are associated with peptidase and hydrolase activities.

We also examined modules that were correlated with the incompatible interaction, displaying expression profiles associated with a failure to cause expanding lesions (Fig. 3). The “dark green” module (1,604 transcripts) is typified by generally stable *in planta* transcript levels that increase at 24 hpi exclusively in the incompatible interaction. The genes in this module are enriched for GO terms related to gene expression, chromatin organization, splicing, mitotic cell cycle, cell division, and DNA repair. The “dark turquoise” module (1,811 transcripts) is characterized by high *in planta* transcript level in the absence of yeast extract, particularly at 0 and 24 hpi, with a steep decline at 12 and 16 hpi, while the expression in the presence of yeast extract remains more stable at all time points. This module contains six genes encoding light-dependent transcription factors (TFs), several light sensors, proteins from the Velvet complex, both phospholipase C proteins, as well as five polyketide synthases. The “light yellow” module (2,504 transcripts) is marked by increased *in planta* transcript levels in the absence of yeast extract at 24 hpi, which are higher in the mutant than in the WT. The only enriched GO terms for this module are related to protein translation. The “light green” module (372 transcripts) is typified by a decline of *in planta* transcript levels over time in the three compatible interactions, while the expression increases at 24 hpi in the incompatible interaction. This module contains RNA polymerase I transcription initiation factors, two HHK histidine kinases, and other signaling proteins, bicarbonate transporters, and other H+ antiporters. The “cyan” module (867 transcripts) is characterized by a peak in expression at 12 hpi, which drops at 16 hpi. Only in the inoculation medium with yeast extract, transcript levels continue to decrease at 24 hpi. This module contains proteins of the proteasome complex, ATPase complex, as well as proteins involved in cytoskeleton binding, glycosylation, and Golgi vesicle transport. The “firebrick4” module (37 transcripts) is typified by stable *in planta* transcript levels that increase at 24 hpi exclusively in the *∆bot2∆boa6#6* mutant without yeast extract, leading to an incompatible interaction. The module contains five copper transporter genes, a copper-binding protein, and a copper metallochaperone, suggesting that the fungus experiences a depletion of copper during the incompatible interaction.

### Overexpressing single CDIPs cannot restore the pathogenicity of *∆bot2∆boa6*

**Fig 5:**
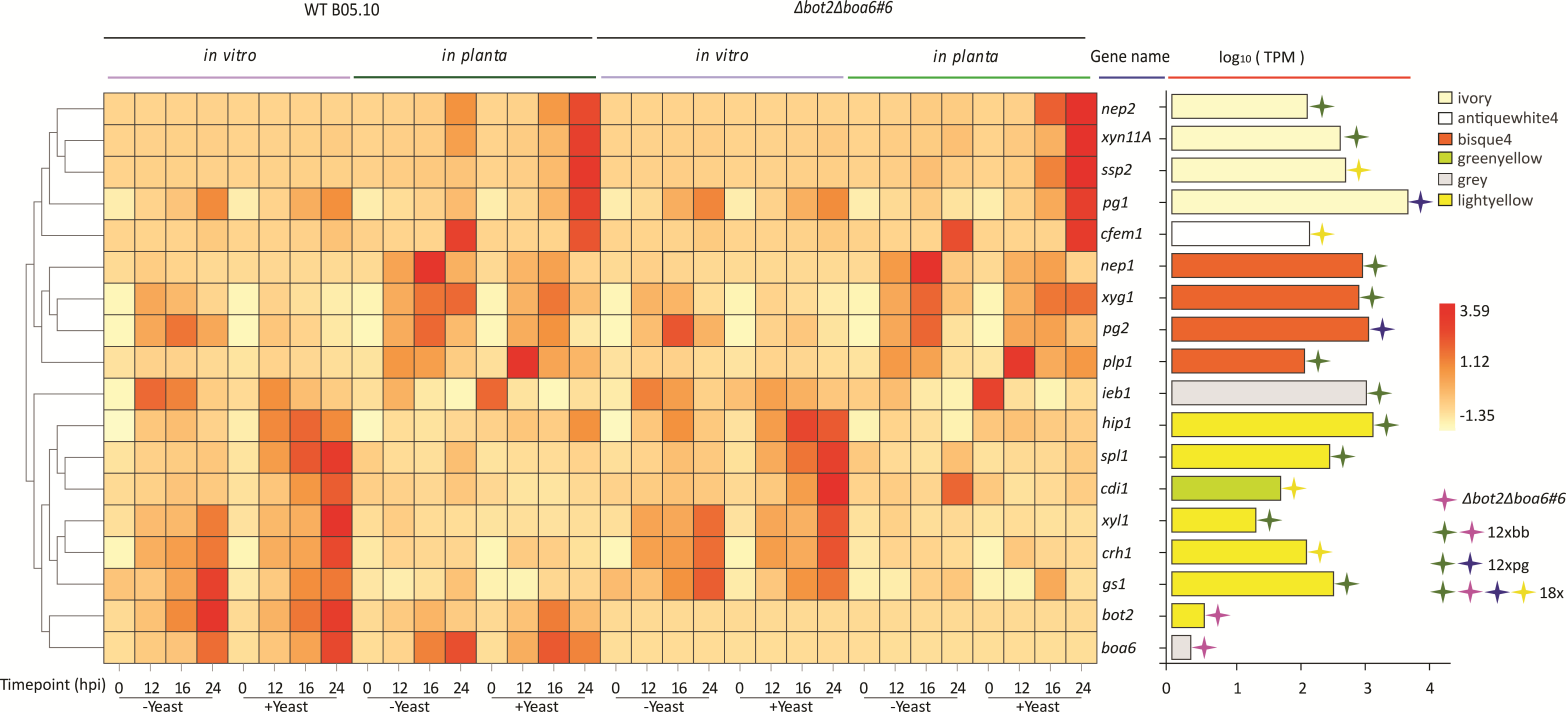
Transcript levels of 18 genes in the WT B05.10 and *∆bot2∆boa6#6*, represented by a heatmap generated by Z-score calculation (left) and the mean log_10_ (TPM) of the gene across all timepoints (right) both during *in vitro* growth and during infection. The genes knocked out in *∆bot2∆boa6#6*, 12xbb, 12xpg, and 18x mutants were marked by plus signs with corresponding colors, for which the legend is at the right bottom corner of the figure. The bar in the mean log_10_ (TPM) panel is filled with a color representing the module in which the gene grouped according to the WGCNA analysis.

Based on phenotypic observations of the mutants in Fig. 1, and the presence of CDIPs in co-expression modules positively correlated with a compatible interaction, we closely examined the expression profiles of CDIP-encoding genes that are deleted in the 18x mutant. A heatmap was generated to compare transcript levels in WT B05.10 and the *∆bot2∆boa6#6* mutant, both *in vitro* and during leaf infection, in the absence or presence of yeast extract (Fig. 5).

Expression patterns of all 16 genes except for *Bcbot2* and *Bcboa6* were similar between WT B05.10 and *∆bot2∆boa6#6*. Notably, *Bcssp2*, *Bcnep2*, *Bcxyn11A,* and *Bcpg1*, members of the “ivory” module, shared a similar transcript profile characterized by their upregulation during tomato infection upon the addition of yeast extract to the inoculum. *Bcssp2* was of particular interest, as it is one of four genes deleted in the 18x mutant but not in the 12xbb or 12xpg mutants, while *Bcnep2*, *Bcxyn11A,* and *Bcpg1* were deleted in the 12xpg mutant (Fig. 5). Since the 12xpg mutant was more virulent than *∆bot2∆boa6#6* in the absence of yeast extract and displayed similar virulence as *∆bot2∆boa6#6* in the presence of yeast extract (Fig. 1), we hypothesized that *Bcssp2* might play a more prominent role in virulence under these conditions than *Bcnep2*, *Bcxyn11A,* or *Bcpg1*. Among the genes deleted specifically in the 18x mutant (but not the 12x mutants), *Bcssp2* was the only gene that was upregulated by addition of yeast extract (Fig. 5); the transcript level of *Bccfem1* remained similar with or without yeast extract, while *Bccdi1* and *Bccrh1* transcripts were downregulated when yeast extract was added.

**Fig 6:**
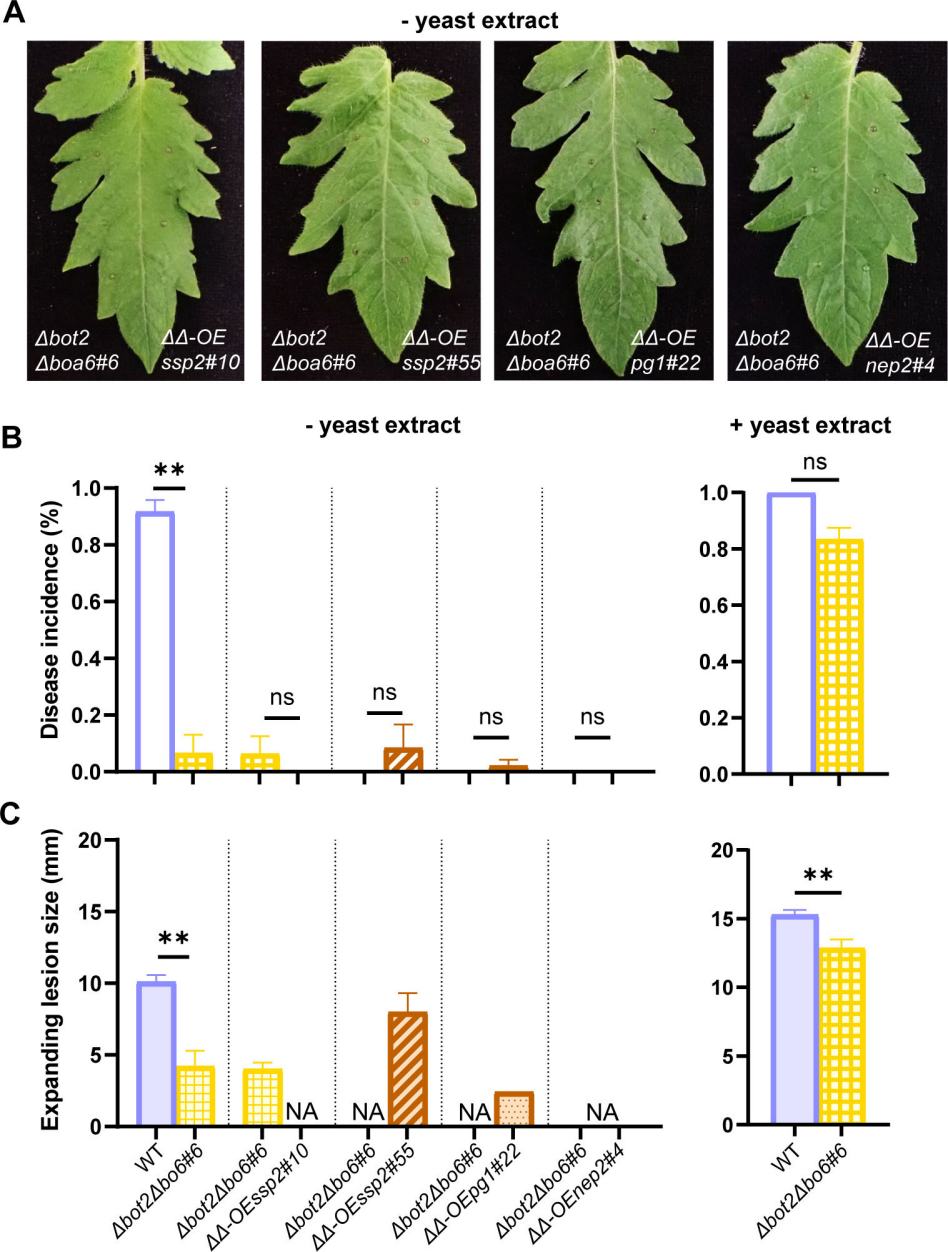
Infection assays to compare the virulence of overexpression mutants to the recipient strain *∆bot2∆boa6#6* on tomato leaves using a Gamborg B5 medium without yeast extract. The comparisons between WT B05.10 and *∆bot2∆boa6#6* were also inoculated using both medium without and with yeast extract in the same experiments, showing similar results as shown in Fig. 1. (**A**) Symptoms of tomato leaflets infected by each overexpression mutant strain (on the right of the central vein) compared with *∆bot2∆boa6#6* (on the left of the central vein) were photographed at 3 dpi. (**B**) Bar charts of disease incidences of inoculations performed using the medium without yeast extract (left) and the disease incidences of WT B05.10 and *∆bot2∆boa6#6* inoculated with the medium with yeast extract in the same experiments (right), which are represented as means with standard errors from 48 datapoints collected from two independent experiments. (**C**) Bar charts representing expanding lesion sizes upon inoculations using the medium without yeast extract (left) and with yeast extract (right), measured by a digital caliper at three dpi. The total number of expanding lesions caused by each of the mutant strains, which were inoculated with the medium without yeast extract, was no more than five from these two experiments. The inoculations of mutant strains causing no expanding lesion are indicated as “NA” in the chart on the left. Statistical analyses were performed by *t*-test, showing no significance (ns) or significant difference (**P < 0.01) in disease incidence and expanding lesion size between the mutant and recipient strain (*∆bot2∆boa6#6* for overexpression mutants or WT B05.10 for *∆bot2∆boa6#6* mutant).

To assess whether overexpression of *Bcssp2* in the *∆bot2∆boa6#6* mutant could restore virulence without addition of yeast extract, by compensating for the absence of BOT and BOA production, fungal transformants were generated, using the *∆bot2∆boa6#6* mutant as a recipient, with *Bcssp2* under control of a constitutive promoter. The virulence of *∆bot2∆boa6-OEssp2* (*∆∆-OEssp2*) mutants was compared with the recipient using inoculation medium lacking yeast extract. Although the potential contribution of *Bcnep2*, *Bcxyn11A,* and *Bcpg1* to the virulence of *B. cinerea* was expected to be less significant than *Bcssp2*, these genes were grouped in the same module based on their expression profile. Therefore, transformants were also generated to separately overexpress either *Bcnep2*, *Bcxyn11A,* or *Bcpg1* in the *∆bot2∆boa6#6* recipient (Fig. S9). Unexpectedly, *∆∆-OExyn11A* transformants showed growth retardation during *in vitro* growth and were eliminated from infection assays. Two transformants of *∆∆-OEssp2*, one transformant of *∆∆-OEpg1*, and one for *∆∆-OEnep2* were inoculated on tomato leaves in medium lacking yeast extract to compare their virulence with the recipient strain *∆bot2∆boa6#6*. None of the overexpression transformants caused a higher proportion of expanding lesions than *∆bot2∆boa6#6* when inoculated without yeast extract (Fig. 6). Thus, overexpressing *Bcssp2, Bcnep2,* or *Bcpg1* individually cannot compensate for the defect in pathogenicity of the *∆bot2∆boa6#6* mutant.

### Expression analysis of plant genes involved in response to pathogens

Despite the fact that our focus was on the fungal transcriptome, we analyzed the transcript level of tomato genes that participate in response to fungal pathogens in order to get insight into how inoculation conditions that result in compatible or incompatible interactions impacted on the host plant expression. The selected tomato gene set included 160 genes involved in phytohormone biosynthesis, perception or response, as well as pathogen-induced transcription factor families and pathogenesis-related proteins (Data S2).

Although the selected gene sets had a very diverse expression pattern across timepoints (Data S2), the heatmap (Fig. S10) looked quite similar between inoculations with WT B05.10 or the *∆bot2∆boa6#6* mutant, and between inoculations with or without yeast extract. Only a few genes in the selected set displayed a more than twofold difference in transcript levels (Data S2). Several ethylene biosynthetic genes and ethylene response factors, as well as two Jasmonate ZIM domain proteins, were induced at 12 and 16 hpi both in the compatible and incompatible interactions, but their transcript levels declined at 24 hpi in an incompatible interaction, while continuing to increase in a compatible interaction. These same genes showed the most pronounced differences in transcript levels at 24 hpi between the inoculations with WT and the *∆bot2∆boa6#6* mutant in medium lacking yeast extract (resulting in compatible and incompatible interactions, respectively).

## DISCUSSION

### The occurrence of an incompatible interaction between a necrotrophic fungus and its host

Most fungal pathologists will by definition consider a gene to encode a virulence factor if a knockout mutant strain produces lesions with significantly smaller lesion size as compared to the wild type. This definition of a virulence factor, however, neglects the complexity of environmental and host influences on pathogen gene expression, growth, and development. The results of this study illustrate the impact of the composition of inoculation medium on disease incidence resulting from inoculation with *B. cinerea* mutants, but not the wild type. The mutants retained their capacity to cause primary necrotic lesions; however, these primary lesions did not expand beyond the inoculation site, resulting in restriction of fungal outgrowth that reflects an “incompatible interaction.” The terms “compatible” and “incompatible” interactions are commonly used for interactions of plants with biotrophic microbial pathogens (21). In such cases, the outcome of an interaction is often determined by gene-for-gene interactions of pathogen effectors, acting as avirulence factors, with cognate receptors in the host plant that recognize the effector and trigger hypersensitive cell death and resistance (22, 23). We are aware that it appears unusual to use the term (in)compatible for the infection of a necrotrophic fungus, and especially in the context of a fungal mutant in combination with the composition of the inoculation medium. We recently adopted the same terminology to describe the interaction between *B. cinerea* WT strain B05.10 and the wild tomato relative *Solanum habrochaites*, in which the sucrose concentration in the inoculum markedly affected disease incidence (24).

In our experiments with *∆bot2#1* and *∆bot2∆boa6#6* mutants, as well as the 12xbb and 18x mutants, the addition of yeast extract to the inoculum restored disease incidence to the level of the WT B05.10 (“compatible interaction”) although lesion sizes caused by the mutants at three dpi were smaller as compared with WT B05.10. The results from virulence assays using both media indicated a more pronounced contribution of BOT and BOA to the virulence of *B. cinerea* than previously reported (7, 13). Leisen et al. (7) reported that the lesion size caused by the *∆bot2∆boa6#6* mutant was similar to that of WT B05.10, using the same Gamborg B5 medium as in this study (without yeast extract) but using for the infection assay a lower spore density combined with a larger droplet size (the total number of conidia in each droplet was similar between both studies). We also observed that the *∆bot2∆boa6#6* mutant caused expanding lesions on tomato leaves using PDB as inoculation medium (Fig. S1B), which differs in the type and concentration of nutrients from Gamborg B5 media (-y/+y). We are not aware of any previous study on *B. cinerea* or any other fungus where such seemingly small variation in experimental procedures leads to opposite outcomes of disease development (incompatible versus compatible interaction). Hopefully, these observations will encourage the research community to more thoroughly consider the methodology used for testing the virulence of knockout mutants in *B. cinerea* and possibly other fungi.

These results inspired us to perform an RNA-seq strategy to acquire insights into the “compatible” or “incompatible” interaction of the *∆bot2∆boa6#6* mutant (but not WT B05.10) with tomato Moneymaker leaves and into the effect of adding yeast extract to the inoculum. We considered that restoration of compatibility for the *∆bot2∆boa6#6* mutant by the addition of yeast extract could be mediated by its influence on the transcript levels of fungal genes that govern crucial stages of the infection, either the primary lesion induction or the subsequent lesion expansion. In order to study these processes with as few experimental variables as possible, we performed an RNA-seq experiment using two media (-/+ yeast extract) as the only difference in the inoculation condition to investigate the molecular mechanisms mediating the transition from an incompatible to a compatible interaction. The focus of our analysis was on *B. cinerea* gene expression in three (early) phases of the infection: penetration of the host surface (12 hpi), initiation of primary cell death (16 hpi), and onset of lesion expansion (24 hpi).

### The role of yeast extract

So, what could be the physiological impact of yeast extract on a fungus during the infection process on a host plant? Yeast extract is a concentrate of the water-soluble portion of autolyzed *Saccharomyces cerevisiae* cells and provides water-soluble vitamins, amino acids, and small peptides to the medium formulation. Gamborg B5 medium consists of minerals, vitamins, and it contains both nitrate and ammonium as nitrogen sources but no carbon source. We supplemented the medium with 25 mM glucose as the carbon source. The Gamborg B5 medium with glucose suffices to allow *B. cinerea* germination, and it enables the WT strain B05.10 to achieve primary lesion induction and subsequent expansion of the lesion (“compatible interaction”) in the absence of supplemented yeast extract. However, yeast extract provides amino acids that enable the fungus to accelerate protein biosynthesis, as it sidesteps the requirement to first synthesize an entire set of amino acids from primary nitrogen and carbon sources in the medium. The immediate availability of (a small amount of) amino acids would give *B. cinerea* a head start that can accelerate the production of proteins and metabolites by a few hours, which stimulates host penetration and the subsequent induction of a primary lesion. Although the WT B05.10 did not require yeast extract to achieve a high disease incidence, the addition of yeast extract to the inoculum resulted in a larger lesion size at three dpi (Fig. 1), presumably facilitated by faster, or more effective, activation of pathogenicity-related mechanisms and thereby enabled the fungus to proceed to the lesion expansion phase at an earlier timepoint. When WT B05.10 was inoculated in Gamborg B5 medium lacking yeast extract, the development of primary lesions was commonly observed from 16 hpi onwards, but expansion of primary lesions occurred only beyond 24 hpi. The addition of yeast extract accelerated the onset of expansion, as the lesions were clearly larger than the inoculation droplet at 24 hpi. *In vitro* tests with medium containing yeast extract or a mixture of amino acids showed that yeast extract indeed accelerated *B. cinerea* conidial germination, but this was not observed upon addition of amino acids only. Moreover, both yeast extract and amino acids reduced or delayed the production of appressoria that function in the penetration of the host surface. Therefore the physiological mechanisms that underlie the positive impact of adding yeast extract to the inoculum on the disease incidence remain elusive.

### Primary lesion induction and lesion expansion are distinct processes

We propose that successful infection by *B. cinerea* involves two stages, which may be governed by distinct fungal virulence factors and which require that the fungus achieves certain milestones at specific time points. It is not merely sufficient to trigger plant cell death by producing a cocktail of CDIMs, but these must be produced and operate timely to prevent the host plant from mounting defense responses that otherwise restrict fungal outgrowth. In this context, it is important to note that the primary lesions that were produced in the incompatible interaction by the *∆bot2∆boa6#6* mutant remained unable to expand once the restriction of lesion outgrowth was observed, regardless of how long incubation was continued. Apparently, the plant response that restricted expansion provided an effective, absolute host resistance response which was achieved around the 24 hpi benchmark. We thus hypothesize that the lack of BOT and BOA production caused such significant delay in the capacity of the fungus to induce host cell death in a timely manner that it allowed the plant to mount an effective resistance. This highlights the importance of proper timing in the outcome of the interaction.

*B. cinerea* expresses different genes at the stages of primary lesion development and lesion expansion. One example is provided by the family of cell death-inducing NLP proteins that contains two members, BcNEP1 and BcNEP2. *Bcnep1* is expressed from 8 hpi onward and reaches a peak at 12–16 hpi, coinciding with the appearance of primary lesions (25). *Bcnep1* transcript levels decline sharply at 24 hpi, coinciding with a strong increase in *Bcnep2* transcript levels, which remain high while lesions continue to expand (25). The cell death-inducing capacity of BcNEP1 protein is stronger than that of BcNEP2 and is notably faster (4–8 h after protein infiltration) than for BcNEP2 (~24 hpi) (25, 26). These results support the hypothesis that primary lesion induction and lesion expansion are distinct processes, involving distinct fungal proteins. In addition, You et al. (27) reported that during infection on tomato, *B. cinerea* expresses the *BcTom*1 gene encoding a ß-xylosidase that hydrolyzes the glycoalkaloid α-tomatine and thereby inactivates its antifungal activity. The expression of *BcTom*1 is low in the absence of α-tomatine and rapidly induced as soon as the fungus comes into contact with α-tomatine. As α-tomatine is located inside vacuoles, contact of *B. cinerea* with α-tomatine only occurs when host cells are damaged by cell death induction. The transcript level of the *BcTom*1 gene can thus serve as a marker for the timing of host cell death in the *B. cinerea*-tomato interaction (28). The transcript profile of *BcTom*1 in this study displayed a different timing between WT and *∆bot2∆boa6#6* mutant and between inoculation media (Table 2).

**TABLE 2:** Normalized expression of *BcTom1* (mean TPM per treatment) across all sequencing samples

WT B05.10								*∆bot2∆boa6#6* double knockout mutant							
t = 0		t = 12		t = 16		t = 24		t = 0		t = 12		t = 16		t = 24	
-y	+y	-y	+y	-y	+y	-y	+y	-y	+y	-y	+y	-y	+y	-y	+y
12	18	12	82	73	362	190	199	8,5	11	12	52	53	260	60	199

In the absence of yeast extract, both WT and *∆bot2∆boa6#6* mutant at 12 hpi showed similar *BcTom*1 transcript levels (TPM values) that were equal to the 0 hpi level, suggesting that the fungus was not yet exposed to α-tomatine. When yeast extract was added, *BcTom*1 expression already increased at 12 hpi by seven- and fourfold in the WT and *∆bot2∆boa6#6* mutant, respectively (Table 2). This observation indicates that host cell death was induced, and primary lesion development was in progress and possibly was more advanced in the WT than in the *∆bot2∆boa6#6* mutant. *BcTom*1 transcript levels continued to increase at subsequent time points. The transcript level in the inoculation without yeast extract at 16 hpi was similar to the level in the inoculation with yeast extract at 12 hpi, both for the *∆bot2∆boa6#6* mutant and WT (Table 2), suggesting that the addition of yeast extract accelerated the host cell death induction by approximately 4 h. At 24 hpi, profiles of the two inoculation conditions diverged notably. *BcTom*1 transcript level upon inoculation of the *∆bot2∆boa6#6* mutant without yeast extract was similar between 16 and 24 hpi, while it more than doubled for the WT under the same conditions. By contrast, transcript levels for inoculation of the *∆bot2∆boa6#6* mutant and the WT inoculated with yeast extract were similar at 24 hpi. In all, these results indicate that yeast extract helped the fungus in accelerating host cell death induction for both *∆bot2∆boa6#6* mutant and WT alike, which therefore advanced the timing of the lesion expansion stage. The shorter primary lesion induction stage might have led to successful lesion expansion by the *∆bot2∆boa6#6* mutant when yeast extract was added, as the host was likely unable to activate effective resistance in such a short period of time.

### Attempts to restore a compatible interaction by overexpression of CDIPs

Neither of the four CDIP-encoding genes tested (*Bcssp2, Bcnep2, Bcxyn11A*, or *Bcpg1*) could, individually, restore the virulence of *∆bot2∆boa6#6* in the absence of yeast extract, which can be explained by three hypotheses. First, additional single (as yet unknown) CDIMs from the ivory module are involved in stimulating primary lesion expansion and thereby overcome the lack of BOT and BOA production. Second, multiple previously described CDIPs might jointly restore pathogenicity of the *∆bot2∆boa6#6* mutant via synergistic action. To validate this hypothesis, it may be useful to generate overexpression mutants in which multiple genes are overexpressed, either by using strong constitutive promoters or by identifying a TF that specifically governs the expression of genes in the ivory module and generating a transformant expressing a constitutively active form of such TF. At present, it is unclear which TF(s) regulate the yeast inducible expression of CDIPs, but recent studies on *B. cinerea* transcriptional networks (29) may help identify TFs that control the expression of virulence factors. Finally, considering that the successful infection of a host by *B. cinerea* requires milestones to be achieved by the fungus at specific time points, *B. cinerea* should induce host programmed cell death (PCD) at a proper intensity and timing. As proposed in reference 3, the early phase of the *B. cinerea*–host interaction resembles a “biotrophic infection” (8–16 hpi), during which the fungus suppresses PCD without triggering plant defense responses in order to permit the biotrophic, pre-symptomatic colonization by the fungus. Therefore, the fungus may require a subtle regulation of PCD during a specific time frame to help *∆bot2∆boa6#6* break through the critical point and initiate the lesion expansion phase. When overexpressing a gene with a strong promoter (e.g., the oliC promoter used in this study), the gene would be expressed at high levels from the germination of conidia onwards. This could lead to CDIP accumulation during the “biotrophic stage” of the fungus, which would prematurely induce host PCD and restrict the invasion of *B. cinerea*. In order to test this hypothesis, we could overexpress gene(s) encoding PCD-inducing molecules in the WT B05.10 background and check whether these transformants trigger host resistance and result in inhibition of the invasion of the fungus.

This study illustrated that the outcome of fungal virulence assays can be strongly influenced by a (seemingly trivial) adjustment in inoculation medium. The phenomenon may be one of the causes of differences in results between different laboratories studying the same pathosystem and clearly deserves more attention in future studies.

## MATERIALS AND METHODS

### Plant and fungal materials and their growth conditions

*B. cinerea* strains used and generated in this study (Table 3) were grown and conidia harvested as described (30). *S. lycopersicum* cv. Moneymaker plants were grown as described (30).

**TABLE 3:** *B. cinerea* strains used in this study

*B. cinerea* strain	Recipient	Description	Reference
B05.10		wildtype *B. cinerea* isolate	(16)
*∆bot2#1*	B05.10	*Bcbot2* knockout mutant	(7)
*∆boa6#1*	B05.10	*Bcboa6* knockout mutant	(13)
*∆bot2∆boa6#6* (∆∆)	B05.10	*Bcbot2* and *Bcboa6* double knockout mutant	(7)
12xbb	B05.10	*Bcbot2, Bcboa6, Bcspl1, Bcnep1, Bcnep2, Bcxyn11A, Bchip1, Bcxyg1, Bcplp1, Bcieb1, Bcxyl1*, and *Bcgs1* were knocked out	(7)
12xpg	B05.10	*Bcpg1, Bcpg2, Bcspl1, Bcnep1, Bcnep2, Bcxyn11A, Bchip1, Bcxyg1, Bcplp1, Bcieb1, Bcxyl1*, and *Bcgs1* were knocked out	(7)
18x	B05.10	*Bcbot2, Bcboa6, Bcpg1, Bcpg2, Bcspl1, Bcnep1, Bcnep2, Bcxyn11A, Bchip1, Bcxyg1, Bcplp1, Bcieb1, Bcxyl1, Bcgs1, Bcssp2, Bccfem1, Bccdi1*, and *Bccrh1* genes were knocked out	(18)
*∆∆-OEssp2#10 / #55*	*∆bot2∆boa6#6*	*Bcssp2* was overexpressed with an oliC promoter, independent transformants *#10* and *#55* were tested in this study	This study
*∆∆-OEpg1#22*	*∆bot2∆boa6#6*	*Bcpg1* was overexpressed with an oliC promoter	This study
*∆∆-OEnep2#4*	*∆bot2∆boa6#6*	*Bcnep2* was overexpressed with an oliC promoter	This study

### Inoculation assays and *in vitro* cultures of *B. cinerea* for RNA sequencing

The media used were Gamborg B5 + vitamins (Duchefa, Haarlem, NL) and yeast extract (Oxoid, UK). *B. cinerea* conidia were diluted to a density of 1,000/μL in Gamborg B5 media (Gamborg B5, 25 mM glucose, 10 mM potassium phosphate, pH 6.0) either without yeast extract or with 0.1% (w/v) yeast extract. The suspension was pre-incubated for 1 h before being inoculated on plants or *in vitro* cultures. For disease assays, each leaf half of *S. lycopersicum* detached leaves was inoculated with 3–4 droplets, each containing 2 μL of inoculum. Inoculated leaves were incubated in a plastic tray with a transparent lid in the laboratory, and disease symptoms were scored at 3 dpi. The number of expanding lesions (diameter > 2 mm) and non-expanding lesions (diameter ≤ 2 mm) was counted for calculating disease incidence (=number of expanding lesions/total inoculations). Diameters of expanding lesions were measured by a digital caliper and leaves were photographed. Statistical analyses were performed and bar charts generated using GraphPad Prism. Details for statistics and charts are in the legends.

To sample tomato leaves for RNA-seq, the adaxial surface of leaves was inoculated in circular areas, each including five droplets containing 2 μL of inoculum. Four leaflets of one compound leaf were inoculated, and one leaflet was sampled at each time point (t = 0, 12, 16, and 24 hpi). Tomato leaves were mock-inoculated with the -yeast extract medium and collected at 0, 12, 16, and 24 hpi. Eight milliliters of *B. cinerea* conidia suspensions in two media (-/+ yeast extract) were pipetted on glass petri dishes (90 mm) and incubated in the laboratory. Fungal tissue was sampled at 0, 12, 16, and 24 hpi for *in vitro* samples. Three biological replicates were collected for all infected and mock-inoculated tomato leaf samples (Table S1). Two biological replicates were collected for *B. cinerea in vitro* cultures (Table S1). Samples were freeze-dried, RNA was extracted using a Maxwell 16 LEV Plant RNA Kit (Promega).

### Germination assay *in vitro* with yeast extract or amino acids

WT (B05.10) conidiospores were added to Gamborg B5 medium supplemented with 25 mM glucose, 10 mM potassium phosphate (pH = 6.0) at a concentration of 40 spores/μL. To the medium, 0.1% yeast extract (Oxoid, LP0021B) or 0.1% amino acid mix (Synthetic yeast drop-out medium lacking Tryptophan (Sigma-Aldrich Y1876), supplemented with L-Tryptophan (Thermo Scientific) was added as indicated. Spores were pre-incubated in 2 mL medium for 1 h. Then, 50-μL droplets of spores were incubated in eight-well glass chamber slides (Lab-Tek II 8-well chamber slides). After 3.5, 4, 5, and 6 hpi, images were taken using a Nikon 80i light microscope. At 5 and 6 hpi, samples were stained with 0.01 mg/mL wheat germ agglutinin-Alexa 488 (WGA488, Invitrogen) to visualize and count appressoria. Spore germination and appressorium formation were counted using the DotDotGoose (v1.7.1) counting software.

### RNA sequencing and identification of DEGs

Strand-specific libraries of RNA samples were constructed, followed by paired-end sequencing on a DNBseq platform (BGI Tech Solutions, Hong Kong), with a read depth of 20–50 million (Table S1). Mapping and quantifying gene transcripts from RNA-seq reads were performed using Kallisto (v0.44.0) (31), with a 100 bootstrap value. PCA was conducted as transcriptome samples clustered by the ggplot2 package in R. Sleuth (v0.30.0) was used for differential expression analysis (32). DEG analysis was done with default settings, removing genes that have <5 estimated read counts in >47% of all the samples. Genes were considered differentially expressed if they displayed between two samples a log2 fold change ≥2 or ≤−2 with an adjusted *P*-value ≤ 0.05 (Benjamini–Hochberg method). UpSet plots were generated using R package UpSetR (33).

### Network construction and co-expression analysis

A total of 80 samples were used for constructing co-expression analysis using R package WGCNA (v1.69) (34). The normalized gene expression matrix was imported into WGCNA to construct co-expression modules. The gene expression matrix was searched for a suitable soft threshold to build gene networks using a scale-free topology model (35). The scale-free network obtained by power processing at β = 13 resulted in an adequate fit with r^2^ = 0.85 with average connectivity approaching 0. Therefore, β = 13 was used to construct a scale-free network. The adjacency matrix was transformed into a topological overlap matrix (TOM) to evaluate the correlation between expression profiles of genes (35). The dissimilar topological matrix (dissTOM= 1 TOM) was used to carry out matrix clustering and module partitioning by the dynamic shearing algorithm. The minimum number of elements in a module was 30 (minModule Size=30), and the threshold for merging of a similar module 0.25 (CutHeight = 0.25) (Fig. S6). Module eigengenes (MEs) were used to calculate correlation coefficients to traits to identify the biologically significant modules. The Pearson correlation coefficient of the corPvalueStudent () function was used to calculate correlations between the infection and yeast extract matrix and the module feature gene matrix to obtain *P*-values. Both a positive and a negative correlation can suggest involvement in either response, and we chose thresholds of [r] > 0.30 and *P* < 0.01 as being significant.

### GO analysis of module genes

Genes in a module were mapped to terms in the GO database (http://www.geneontology.org/), and were enriched as compared to the genome background (36). The GO terms were subsequently filtered for the ones belonging to the domains of biological processes and molecular functions. Numbers were calculated for every term, and enriched GO terms in modules were defined by hypergeometric distribution algorithm. FDR was set to a threshold ≤ 0.05. GO enrichment was visualized using ggplot2 (R package) (37).

### *B. cinerea* transformation by CRISPR-Cas9 mediated approach

*B. cinerea* mutant strains were generated by CRISPR-Cas9 mediated transformation with minor modification from (38). Primers for synthesis of sgRNAs and amplification of donor templates are in Table S2. *Bcssp2, Bcpg1,* and *Bcnep2* were amplified from B05.10 genomic DNA by PCR using Phusion Hot Start II DNA Polymerase (Thermo Scientific) and cloned into pNDH-OGG or pNAN-OGG (39) by replacing the GFP cassette using the ClonExpress MultiS One Step Cloning Kit (Vazyme). Donor templates for transformation were amplified from pNDH-OGG harboring *Bcssp2* or from pNAN-OGG vector harboring either *Bcpg1* or *Bcnep2. Bcssp2* was overexpressed by replacing *BcniaD* in the *∆bot2∆boa6#6* recipient strain via homologous recombination upon cleavage of Cas9 at *BcniaD* to obtain *∆∆-OEssp2* transformants, while *Bcpg1* or *Bcnep2* was overexpressed by replacing *BcniiA* to obtain *∆∆-OEpg1* or *∆∆-OEnep2* mutants. Methods for molecular characterization of transformants are described in reference 30 using primers in Table S2.

## Data Availability

The Bioproject is deposited in NCBI under accession PRJNA1173356. The relevant biosample accession numbers are SAMN44302583 and SAMN44302584. Sequence data were deposited in the Sequence Read Archive under accession numbers SRA40446600 - SRA40446681.

## SUPPLEMENTAL MATERIAL

Data S1 mbio.03119-25-s0001.xlsx Genes encoding secreted proteins in three modules, which are positively correlated to the compatible interaction.

Data S2 mbio.03119-25-s0002.xlsx Transcript levels of 160 tomato genes involved in defense responses and phytohormone production, signaling, and response, as well as pathogenesis-related proteins.

Supplemental material mbio.03119-25-s0003.pdf Supplemental figures and tables and captions for Data S1 and S2.

ASM does not own the copyrights to Supplemental Material that may be linked to, or accessed through, an article. The authors have granted ASM a non-exclusive, world-wide license to publish the Supplemental Material files. Please contact the corresponding author directly for reuse.

## References

[1] Elad Y, Pertot I, Cotes Prado AM, Stewart A. 2016. Plant hosts of *Botrytis* spp, p 413–486. In Botrytis - the fungus, the pathogen and its management in agricultural systems. Springer International Publishing.

[2] van Kan JAL. 2006. Licensed to kill: the lifestyle of a necrotrophic plant pathogen. Trends Plant Sci 11:247–253. doi:10.1016/j.tplants.2006.03.005 16616579

[3] Veloso J, van Kan JAL. 2018. Many shades of grey in Botrytis–host plant interactions. Trends Plant Sci 23:613–622. doi:10.1016/j.tplants.2018.03.016 29724660

[4] Collado IG, Sánchez AJM, Hanson JR. 2007. Fungal terpene metabolites: biosynthetic relationships and the control of the phytopathogenic fungus Botrytis cinerea. Nat Prod Rep 24:674–686. doi:10.1039/b603085h 17653354

[5] Reino JL, Durán-Patrón RM, Daoubi M, Collado IG, Hernández-Galán R. 2006. Biosynthetic studies on the botcinolide skeleton: new hydroxylated lactones from Botrytis cinerea. J Org Chem 71:562–565. doi:10.1021/jo051993s 16408965

[6] Collado IG, Viaud M. 2015. Secondary metabolism in *Botrytis cinerea*: combining genomic and metabolomic approaches, p 291–313. In Botrytis - the fungus, the pathogen and its management in agricultural systems. Springer International Publishing.

[7] Leisen T, Werner J, Pattar P, Safari N, Ymeri E, Sommer F, Schroda M, Suárez I, Collado IG, Scheuring D, Hahn M. 2022. Multiple knockout mutants reveal a high redundancy of phytotoxic compounds contributing to necrotrophic pathogenesis of Botrytis cinerea. PLoS Pathog 18:e1010367. doi:10.1371/journal.ppat.1010367 35239739 PMC8923502

[8] Zhang L, Hua C, Pruitt RN, Qin S, Wang L, Albert I, Albert M, van Kan JAL, Nürnberger T. 2021. Distinct immune sensor systems for fungal endopolygalacturonases in closely related Brassicaceae. Nat Plants 7:1254–1263. doi:10.1038/s41477-021-00982-2 34326531

[9] Bi K, Scalschi L, Jaiswal N, Mengiste T, Fried R, Sanz AB, Arroyo J, Zhu W, Masrati G, Sharon A. 2021. The Botrytis cinerea Crh1 transglycosylase is a cytoplasmic effector triggering plant cell death and defense response. Nat Commun 12:2166. doi:10.1038/s41467-021-22436-1 33846308 PMC8042016

[10] Zhu W, Wei W, Wu Y, Zhou Y, Peng F, Zhang S, Chen P, Xu X. 2017. BcCFEM1, a CFEM domain-containing protein with putative GPI-anchored site, is involved in pathogenicity, conidial production, and stress tolerance in Botrytis cinerea. Front Microbiol 8:1807. doi:10.3389/fmicb.2017.01807 28979251 PMC5611420

[11] Denton-Giles M, McCarthy H, Sehrish T, Dijkwel Y, Mesarich CH, Bradshaw RE, Cox MP, Dijkwel PP. 2020. Conservation and expansion of a necrosis-inducing small secreted protein family from host-variable phytopathogens of the Sclerotiniaceae. Mol Plant Pathol 21:512–526. doi:10.1111/mpp.12913 32061186 PMC7060139

[12] Sperschneider J, Gardiner DM, Dodds PN, Tini F, Covarelli L, Singh KB, Manners JM, Taylor JM. 2016. EffectorP: predicting fungal effector proteins from secretomes using machine learning. New Phytol 210:743–761. doi:10.1111/nph.13794 26680733

[13] Dalmais B, Schumacher J, Moraga J, LE Pêcheur P, Tudzynski B, Collado IG, Viaud M. 2011. The Botrytis cinerea phytotoxin botcinic acid requires two polyketide synthases for production and has a redundant role in virulence with botrydial. Mol Plant Pathol 12:564–579. doi:10.1111/j.1364-3703.2010.00692.x 21722295 PMC6640383

[14] Pinedo C, Wang C-M, Pradier J-M, Dalmais B, Choquer M, Le Pêcheur P, Morgant G, Collado IG, Cane DE, Viaud M. 2008. Sesquiterpene synthase from the botrydial biosynthetic gene cluster of the phytopathogen Botrytis cinerea. ACS Chem Biol 3:791–801. doi:10.1021/cb800225v 19035644 PMC2707148

[15] Porquier A, Morgant G, Moraga J, Dalmais B, Luyten I, Simon A, Pradier JM, Amselem J, Collado IG, Viaud M. 2016. The botrydial biosynthetic gene cluster of Botrytis cinerea displays a bipartite genomic structure and is positively regulated by the putative Zn(II)_2_Cys_6_ transcription factor BcBot6. Fungal Genet Biol 96:33–46. doi:10.1016/j.fgb.2016.10.003 27721016

[16] Van Kan JAL, Stassen JHM, Mosbach A, Van Der Lee TAJ, Faino L, Farmer AD, Papasotiriou DG, Zhou S, Seidl MF, Cottam E, Edel D, Hahn M, Schwartz DC, Dietrich RA, Widdison S, Scalliet G. 2017. A gapless genome sequence of the fungus Botrytis cinerea. Mol Plant Pathol 18:75–89. doi:10.1111/mpp.12384 26913498 PMC6638203

[17] Porquier A, Moraga J, Morgant G, Dalmais B, Simon A, Sghyer H, Collado IG, Viaud M. 2019. Botcinic acid biosynthesis in Botrytis cinerea relies on a subtelomeric gene cluster surrounded by relics of transposons and is regulated by the Zn_2_Cys_6_ transcription factor BcBoa13. Curr Genet 65:965–980. doi:10.1007/s00294-019-00952-4 30848345

[18] Müller T, Bronkhorst J, Müller J, Safari N, Hahn M, Sprakel J, Scheuring D. 2024. Plant infection by the necrotrophic fungus Botrytis requires actin-dependent generation of high invasive turgor pressure. New Phytol 244:192–201. doi:10.1111/nph.20025 39107894

[19] Liu N, Lian S, Li B, Ren W. 2022. The autophagy protein BcAtg2 regulates growth, development and pathogenicity in the gray mold fungus Botrytis cinerea. Phytopathol Res 4:3. doi:10.1186/s42483-022-00108-2

[20] Yorimitsu T, Klionsky DJ. 2005. Autophagy: molecular machinery for self-eating. Cell Death Differ 12 Suppl 2:1542–1552. doi:10.1038/sj.cdd.4401765 16247502 PMC1828868

[21] Keen NT. 1990. Gene-for-gene complementarity in plant-pathogen interactions. Annu Rev Genet 24:447–463. doi:10.1146/annurev.ge.24.120190.002311 2088175

[22] Jones JDG, Dangl JL. 2006. The plant immune system. Nature 444:323–329. doi:10.1038/nature05286 17108957

[23] Giraldo MC, Valent B. 2013. Filamentous plant pathogen effectors in action. Nat Rev Microbiol 11:800–814. doi:10.1038/nrmicro3119 24129511

[24] You Y, Astudillo-Estévez I, Essenstam B, Qin S, van Kan JAL. 2023. Leaf resistance to Botrytis cinerea in wild tomato Solanum habrochaites depends on inoculum composition. Front Plant Sci 14:1156804. doi:10.3389/fpls.2023.1156804 37600190 PMC10433766

[25] Cuesta Arenas Y, Kalkman ERIC, Schouten A, Dieho M, Vredenbregt P, Uwumukiza B, Osés Ruiz M, van Kan JAL. 2010. Functional analysis and mode of action of phytotoxic Nep1-like proteins of Botrytis cinerea. Physiol Mol Plant Pathol 74:376–386. doi:10.1016/j.pmpp.2010.06.003

[26] Schouten A, Van Baarlen P, Van Kan JAL. 2008. Phytotoxic Nep1-like proteins from the necrotrophic fungus Botrytis cinerea associate with membranes and the nucleus of plant cells. New Phytol 177:493–505. doi:10.1111/j.1469-8137.2007.02274.x 18028294

[27] You Y, Suraj HM, Matz L, Herrera Valderrama AL, Ruigrok P, Shi-Kunne X, Pieterse FPJ, Oostlander A, Beenen HG, Chavarro-Carrero EA, Qin S, Verstappen FWA, Kappers IF, Fleißner A, van Kan JAL. 2024. Botrytis cinerea combines four molecular strategies to tolerate membrane-permeating plant compounds and to increase virulence. Nat Commun 15:6448. doi:10.1038/s41467-024-50748-5 39085234 PMC11291775

[28] You Y. 2022. Host resistance mechanisms and fungal infection strategies in the Botrytis cinerea-tomato interaction Doctoral dissertation, Wageningen University

[29] Olivares-Yañez C, Sánchez E, Pérez-Lara G, Seguel A, Camejo PY, Larrondo LF, Vidal EA, Canessa P. 2021. A comprehensive transcription factor and DNA-binding motif resource for the construction of gene regulatory networks in Botrytis cinerea and Trichoderma atroviride. Comput Struct Biotechnol J 19:6212–6228. doi:10.1016/j.csbj.2021.11.012 34900134 PMC8637145

[30] Qin S, Veloso J, Baak M, Boogmans B, Bosman T, Puccetti G, Shi-Kunne X, Smit S, Grant-Downton R, Leisen T, Hahn M, van Kan JAL. 2023. Molecular characterization reveals no functional evidence for naturally occurring cross-kingdom RNA interference in the early stages of Botrytis cinerea-tomato interaction. Mol Plant Pathol 24:3–15. doi:10.1111/mpp.13269 36168919 PMC9742496

[31] Bray NL, Pimentel H, Melsted P, Pachter L. 2016. Near-optimal probabilistic RNA-seq quantification. Nat Biotechnol 34:525–527. doi:10.1038/nbt.3519 27043002

[32] Pimentel H, Bray NL, Puente S, Melsted P, Pachter L. 2017. Differential analysis of RNA-seq incorporating quantification uncertainty. Nat Methods 14:687–690. doi:10.1038/nmeth.4324 28581496

[33] Conway JR, Lex A, Gehlenborg N. 2017. UpSetR: an R package for the visualization of intersecting sets and their properties. Bioinformatics 33:2938–2940. doi:10.1093/bioinformatics/btx364 28645171 PMC5870712

[34] Langfelder P, Horvath S. 2008. WGCNA: an R package for weighted correlation network analysis. BMC Bioinformatics 9:1–13. doi:10.1186/1471-2105-9-559 19114008 PMC2631488

[35] Wang N, Wang R, Wang R, Chen S. 2018. Transcriptomics analysis revealing candidate networks and genes for the body size sexual dimorphism of Chinese tongue sole (Cynoglossus semilaevis). Funct Integr Genomics 18:327–339. doi:10.1007/s10142-018-0595-y 29532338

[36] Ashburner M, Ball CA, Blake JA, Botstein D, Butler H, Cherry JM, Davis AP, Dolinski K, Dwight SS, Eppig JT, Harris MA, Hill DP, Issel-Tarver L, Kasarskis A, Lewis S, Matese JC, Richardson JE, Ringwald M, Rubin GM, Sherlock G. 2000. Gene Ontology: tool for the unification of biology. Nat Genet 25:25–29. doi:10.1038/75556 10802651 PMC3037419

[37] Wickham H, Chang W, Wickham MH. 2016. Package ‘ggplot2. 1st ed. Create elegant data visualisations using the grammar of graphics. https://ggplot2.tidyverse.org/reference/ggplot2-package.html.

[38] Leisen T, Bietz F, Werner J, Wegner A, Schaffrath U, Scheuring D, Willmund F, Mosbach A, Scalliet G, Hahn M. 2020. CRISPR/Cas with ribonucleoprotein complexes and transiently selected telomere vectors allows highly efficient marker-free and multiple genome editing in Botrytis cinerea. PLoS Pathog 16:e1008326. doi:10.1371/journal.ppat.1008326 32804988 PMC7451986

[39] Schumacher J. 2012. Tools for Botrytis cinerea: new expression vectors make the gray mold fungus more accessible to cell biology approaches. Fungal Genet Biol 49:483–497. doi:10.1016/j.fgb.2012.03.005 22503771

