# Supplementary material for: Loss of virulence of Botrytis cinerea mutants defective in phytotoxin production is restored by modifying inoculation medium
Source: mBio. 2025 Dec 29;17(2):e03119-25. doi: 10.1128/mbio.03119-25 (PMC12892934; doi:10.1128/mbio.03119-25)
Supplement: Supplemental material — Supplemental figures and tables and captions for Data S1 and S2. [file mbio.03119-25-s0003.pdf]

**Supplementary material to the manuscript**

# "Loss of virulence of *Botrytis cinerea* mutants defective in phytotoxin production is restored by modifying inoculation medium"

Si Qin, Xiaoqian Shi-Kunne, Jie Chen, Frank Pieterse, Henriek Beenen, Yaohua You, Jan van Kan

Laboratory of Phytopathology, Wageningen University, Wageningen, The Netherlands

**Supplementary Figure S1.** Virulence of *B. cinerea* mutants on tomato leaves using different inoculation media.

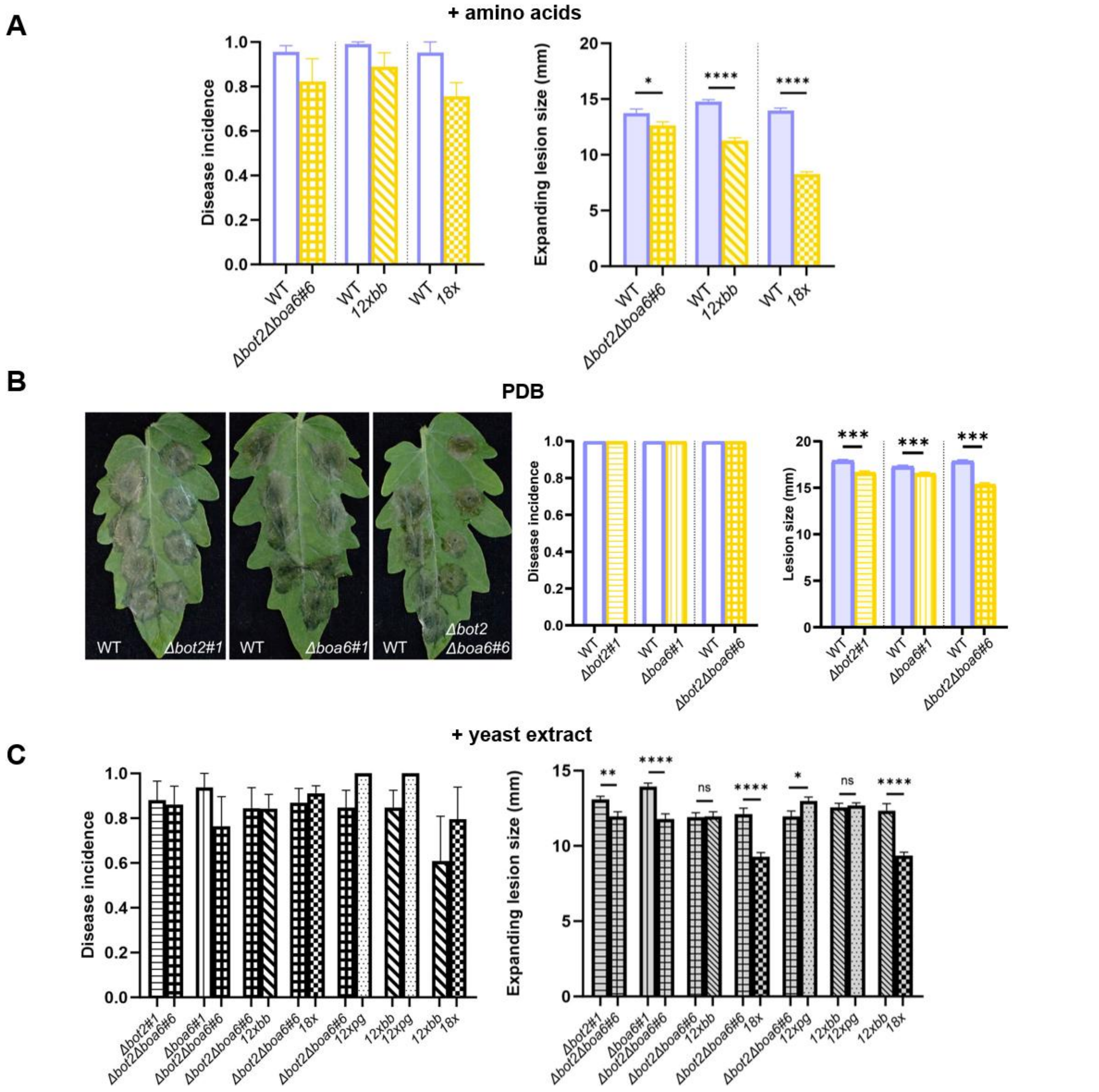


**(A)** Virulence of *Δbot2#1, Δboa6#1* and *Δbot2Δboa6#6* compared with WT B05.10 on tomato leaves, using GB5 medium with amino acids as inoculation medium. Bar charts of disease incidences (left) and lesion sizes (right) at 3 dpi, presented as means with SD from approx. 90 datapoints from 3 experiments. **(B)** Virulence of *Δbot2#1, Δboa6#1* and *Δbot2Δboa6#6* compared with WT B05.10 on tomato leaves, using PDB as inoculation medium. Symptoms on tomato leaves infected by each mutant (right of the central vein) compared with WT B05.10 (left of the central vein), photographed at 3 dpi (left). Bar charts of disease incidences (middle) and lesion sizes (right) at 3 dpi, presented as means with SD from 96 datapoints from 3 experiments. **(C)** Pairwise virulence comparisons between different *B. cinerea* mutants, including *Δbot2#1, Δboa6#1 Δbot2Δboa6#6,* 12xbb, 12xpg and 18x on tomato leaves. These assays were performed using GB5 medium supplemented with yeast extract. Bar charts of disease incidences (left) and lesion sizes (right) at 3 dpi, presented as means with SD from ~90 datapoints from 3 experiments. Two-tailed Student's T-test was used for statistics. There was no significant difference in any pairwise comparison in disease incidence. Asterisks on plots of expanding lesion sizes indicate significant differences (* p<0.05, ** p< 0.01, *** p<0.001 and **** p<0.0001), and "ns" stands for non-significance.

**Supplementary Figure S2.** The effect of yeast extract and amino acids on *B. cinerea* germination and appressorium development.

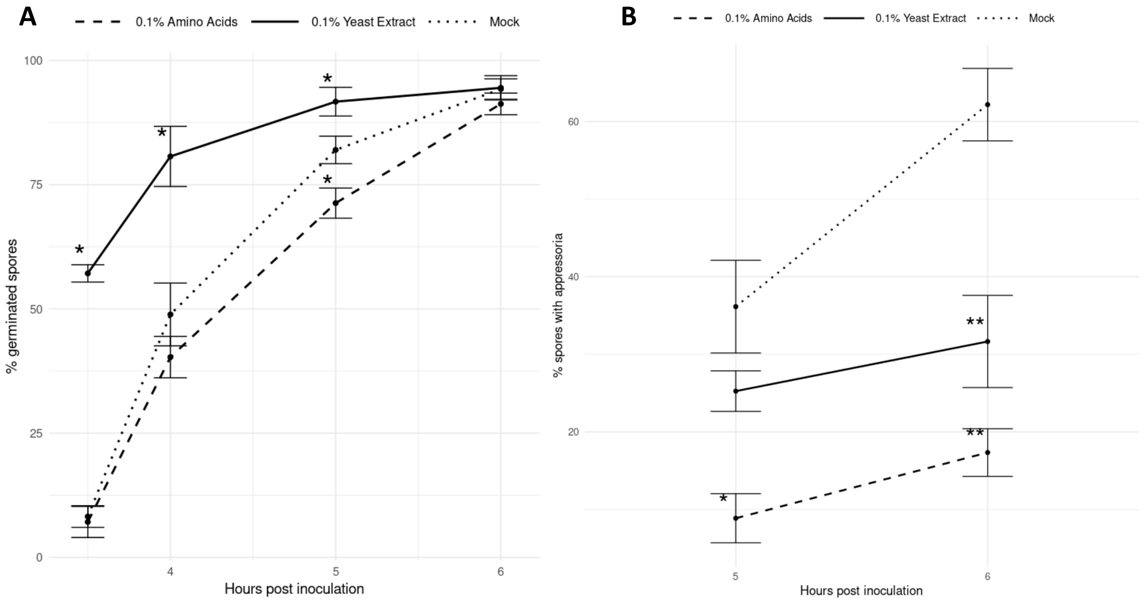


Germination (A) and appressorium development (B) of *B. cinerea* (strain B05.10) conidia over a 6h time range in Gamborg B5 medium with 25mM sucrose and 10mM $KH_2PO_4$ (dotted line, n=4), or in the same medium supplemented with 0.1% yeast extract (solid line, n=6), or supplemented with 0.1% amino acids (20 amino acids in equimolar quantities, dashed line, n=6,). At each time point, significantly differing germination percentages are indicated with (*) asterisks (Mann-Whitney U test, Bonferroni correction, $p<0.05$).

**Supplementary Figure S3** PCA plot of the RNA-seq dataset shows the differences (distances) between all samples generated in this study, according to gene expression levels.

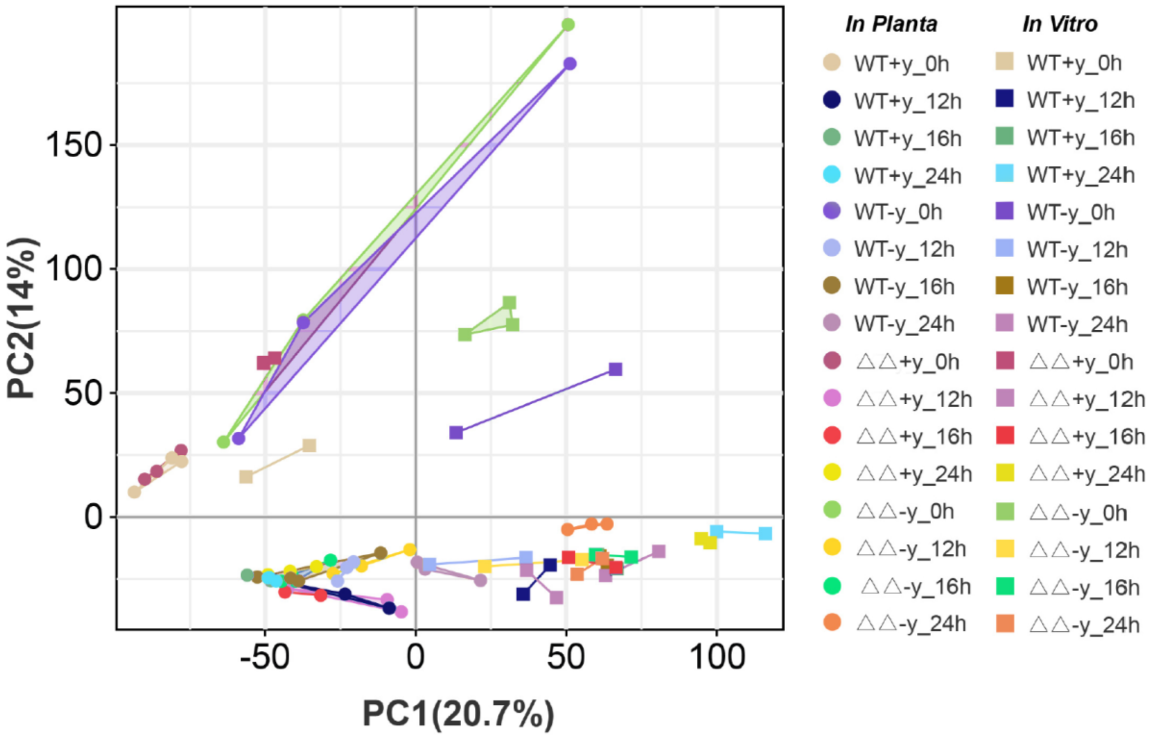


Each round dot indicates an *in planta* sample, and each squared dot indicates an *in vitro* sample. Different colors represent different fungal strains (WT B05.10 and *Δbot2Δboa6#6* (WT and ΔΔ)), different media (- and +y), and different timepoints (0, 12, 16 or 24 hpi).

**Supplementary Figure S4.** GO enrichment analysis

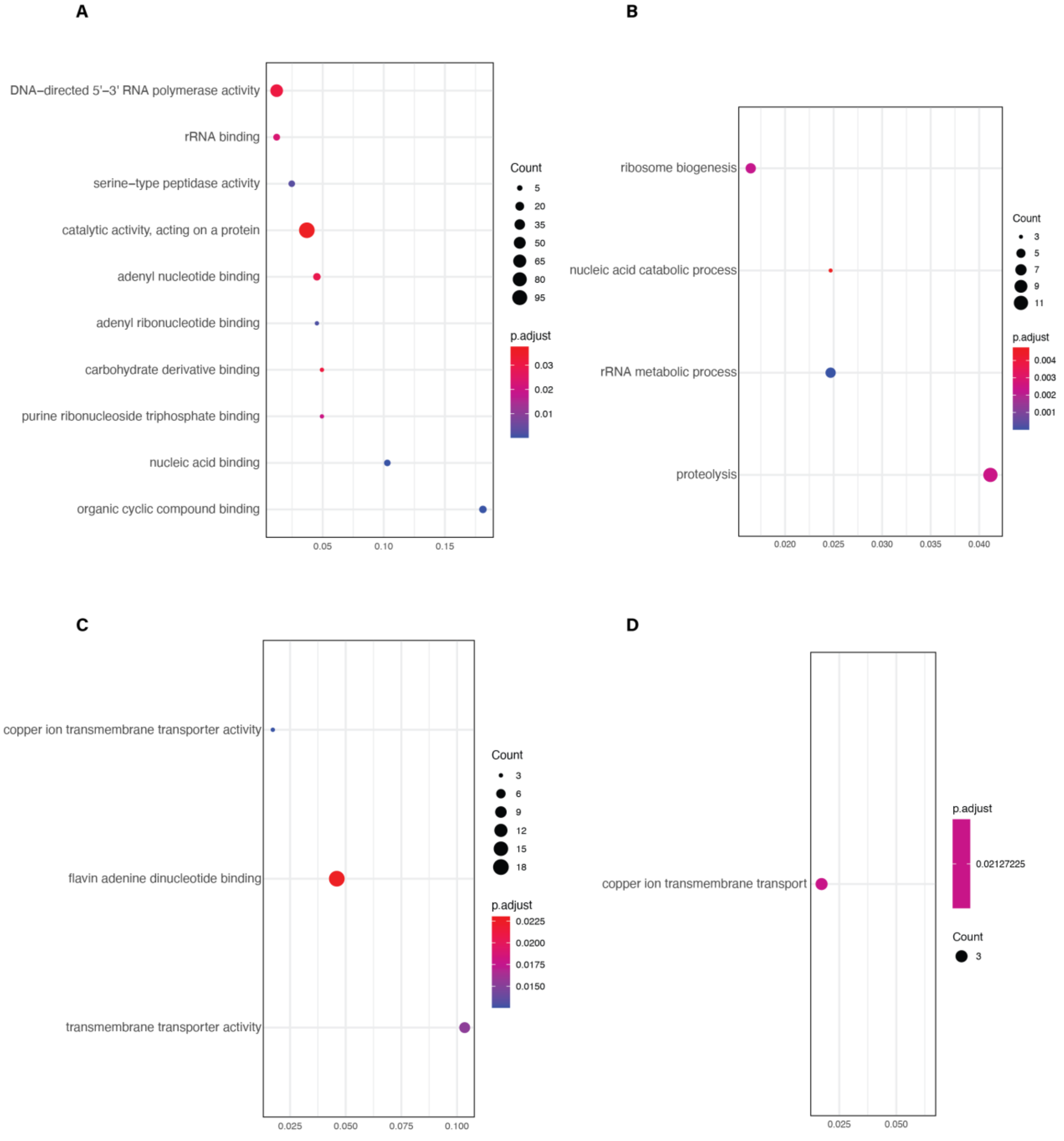


GO enrichment analysis for genes that are up-regulated between +yeast and -yeast (A and B ) and down-regulated (C and D) both in WT and mutant at 24 hpi. (A, C) Molecular function. (B, D) Biological process.

**Supplementary Figure S5.** GO enrichment analysis

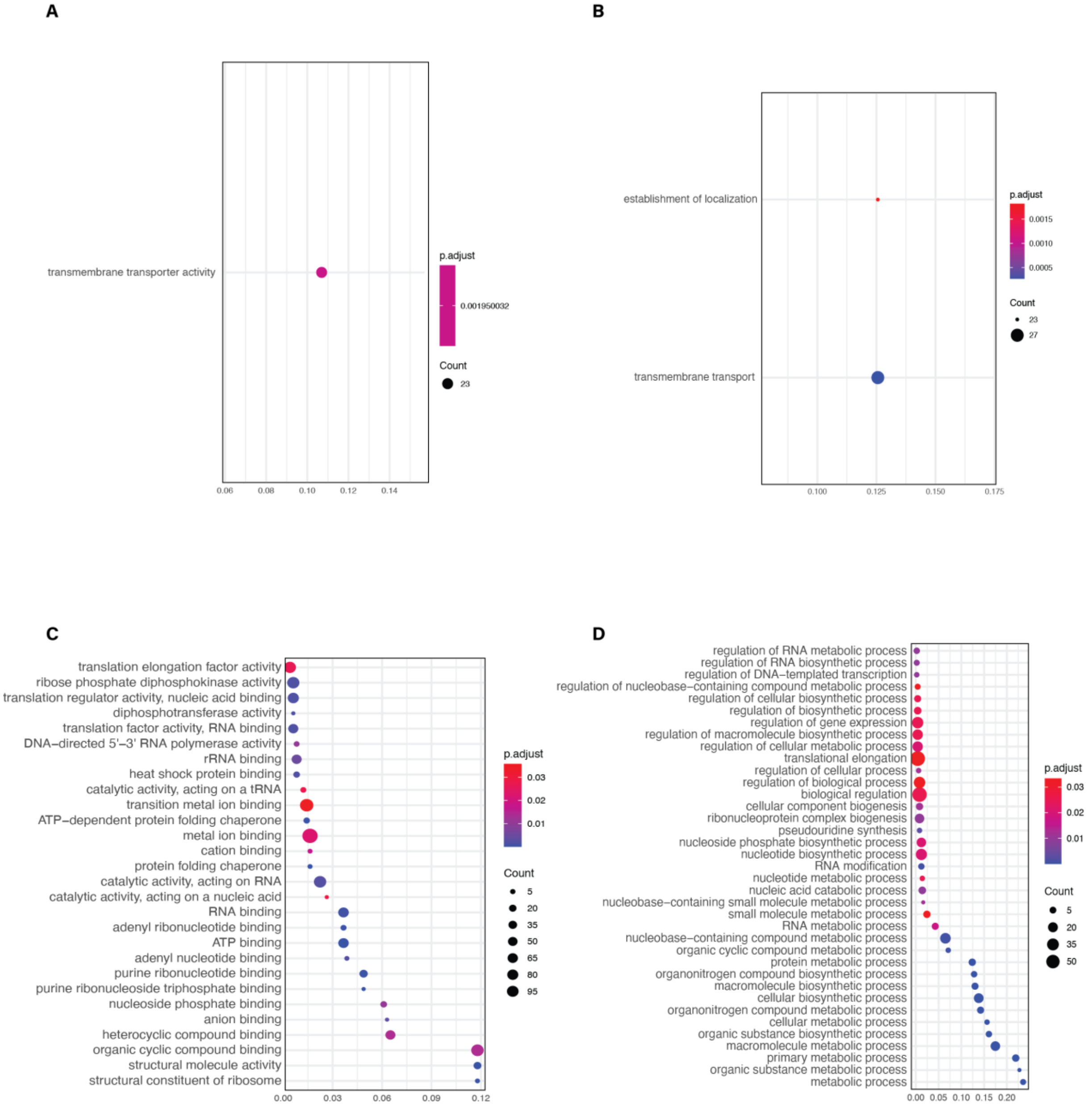


GO enrichment analysis for DEGs (+yeast vs -yeast) that are unique in WT (A, B) and mutant (C, D) at 24 hpi. WT only contains enriched GO terms in down-regulated DEGs (A, B), whereas mutant contains only GO terms in up-regulated genes (C, D). (A, C) Molecular function. (B, D) Biological process.

**Supplementary Figure S6.** Dendrogram of WGCNA gene co-expression network.

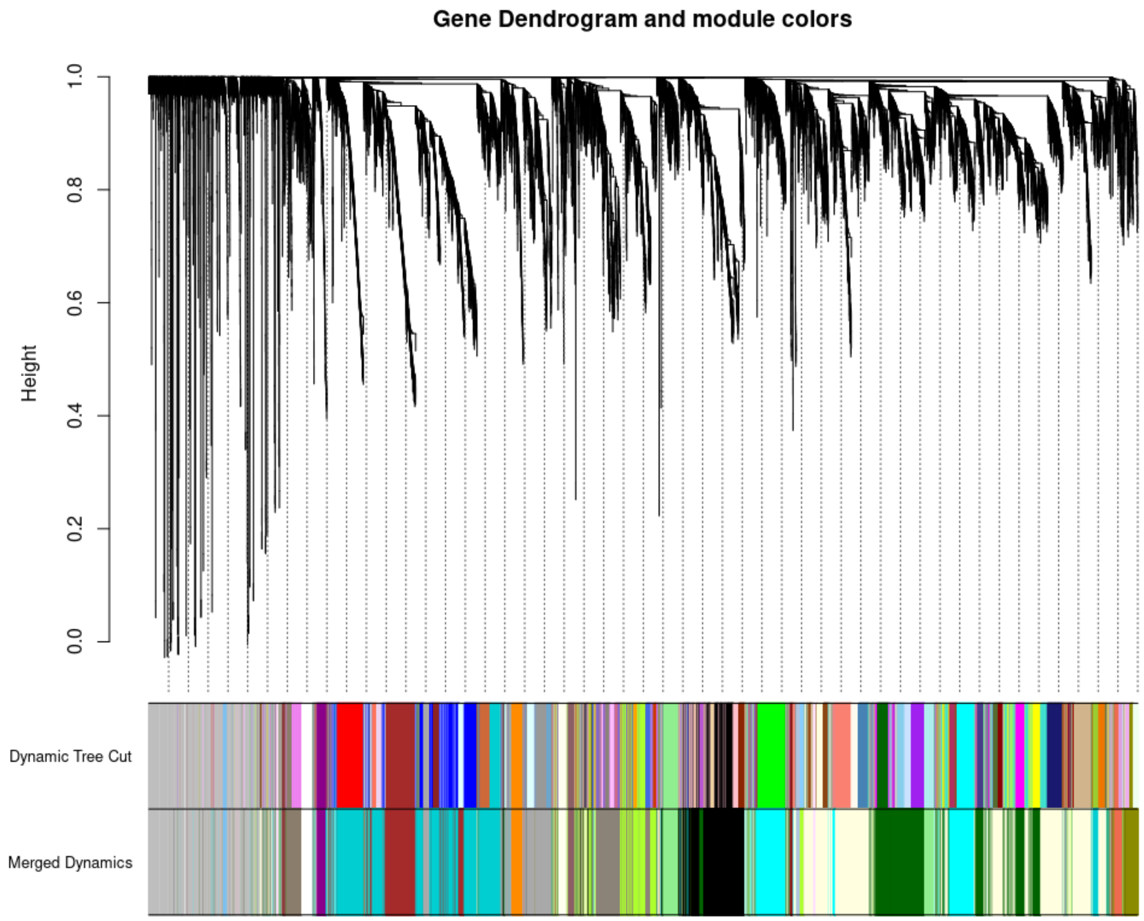


In the top part of the figure, a dendrogram of the filtered set of genes is shown, constructed using the topological overlap between genes. Below the dendrogram, the top colored bar indicates the co-expression modules produced by the dynamic tree cutting algorithm, and the bottom colored bar indicates the merged co-expression modules.

**Supplementary Figure S7**. Module significance correlation based on the Pearson correlation.

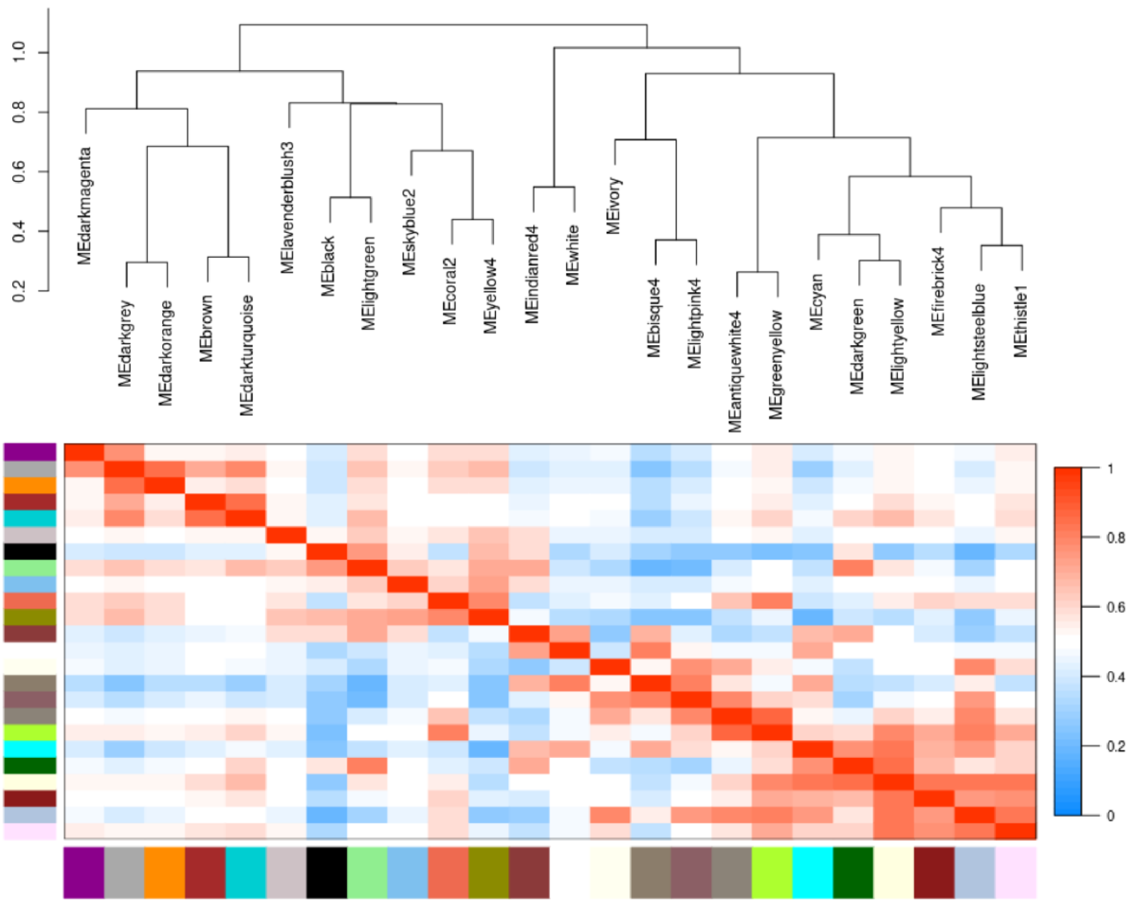

**Supplementary Figure S8.** GO enrichment analysis of three gene modules

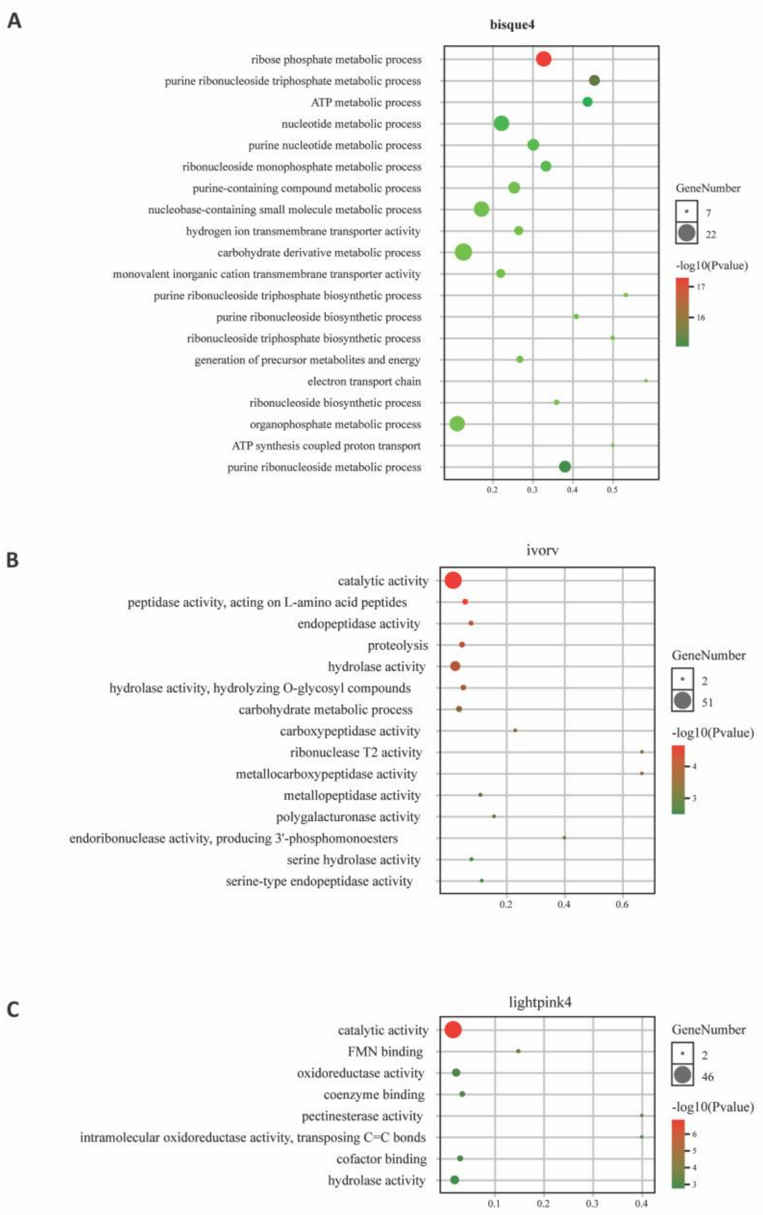


GO enrichment analysis of three *B. cinerea* gene modules of which the expression profile is positively correlated to the compatible interaction between *B. cinerea* and tomato (i.e. the plant becoming diseased).

**Supplementary Figure S9** Molecular characterization of overexpression mutants

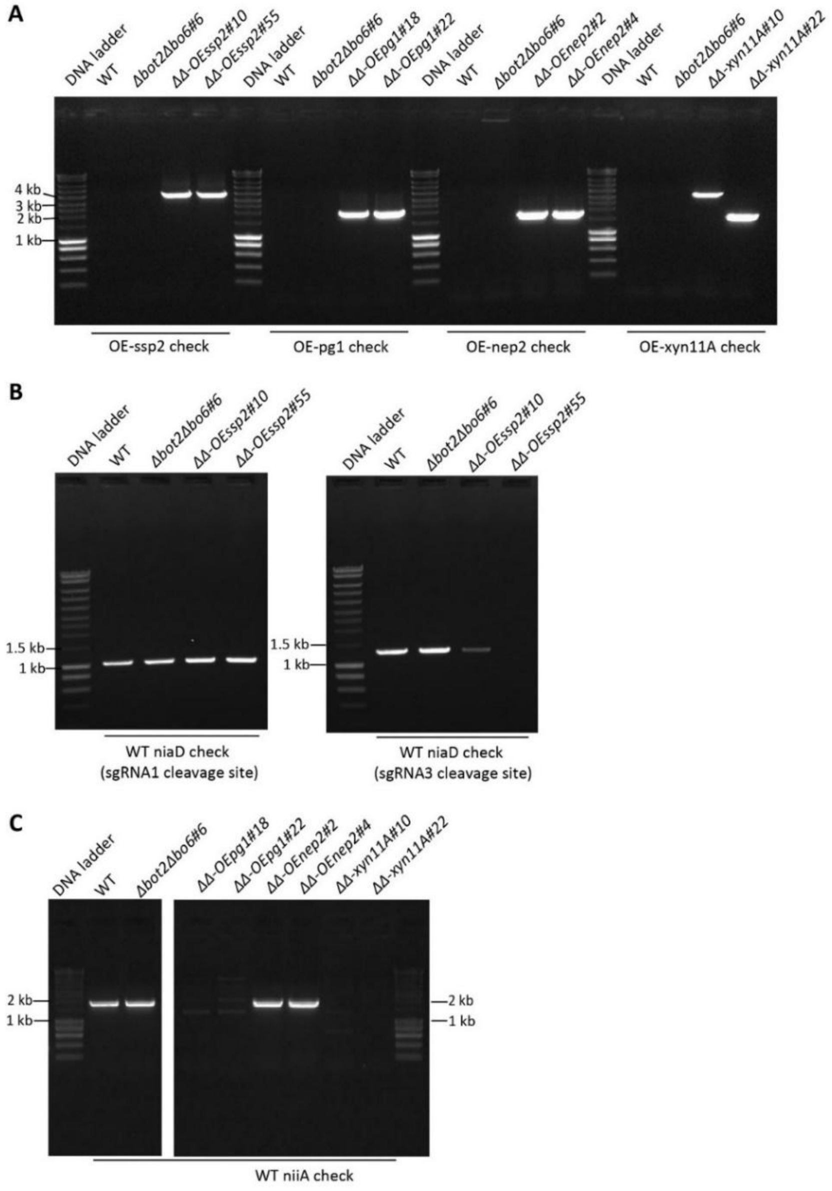


Molecular characterization of overexpression mutants *ΔΔ-OEssp2#10, ΔΔ-OEssp2#55*, *ΔΔ-OEpg1#18*, *ΔΔ-OEpg1#22, ΔΔ-OEnep2#2, ΔΔ-OEnep2#4, ΔΔ-OExyn11A#10* and *ΔΔ-OExyn11A#22*. WT B05.10 and *Δbot2Δboa6#6* double mutant were used as controls. **(A)** PCR results confirmed that integration of the overexpression cassettes for *ssp2*, *pg1*, *nep2* and *xyn11A* were successful. Two independent transformants for each construct were obtained. The donor-templates for the OE*ssp2* and *OExyn11A* transformants were inserted ectopically inside of the *niaD* gene instead of replacing the whole *niaD* coding sequence. A similar strategy was chosen for the. The other transformants were obtained via replacing the entire *niiA* gene by the corresponding overexpression-donor-template. **(B)** The insertion of OE*ssp2*-donor-template by NHEJ was confirmed to be located at the sgRNA3 cleavage site for *ΔΔ-OEssp2#55* which was a homokaryotic transformant. *ΔΔ-OEssp2#10* was either a heterokaryon or the ectopic insertion location cannot be confirmed by these PCR. (C) *ΔΔ-OEpg1#18*, *ΔΔ-OEpg1#22, ΔΔ-OExyn11A#10* and *ΔΔ-OExyn11A#22* were confirmed to be homozygous transformants, while *ΔΔ-OEnep2#2* and *ΔΔ-OEnep2#4* were heterokaryons. Names of the *B. cinerea* strains are indicated above each DNA gel picture, and the aims for the primer combinations are indicated below the gel.

**Supplementary Figure S10.** Heat map of expression of tomato defense-related genes

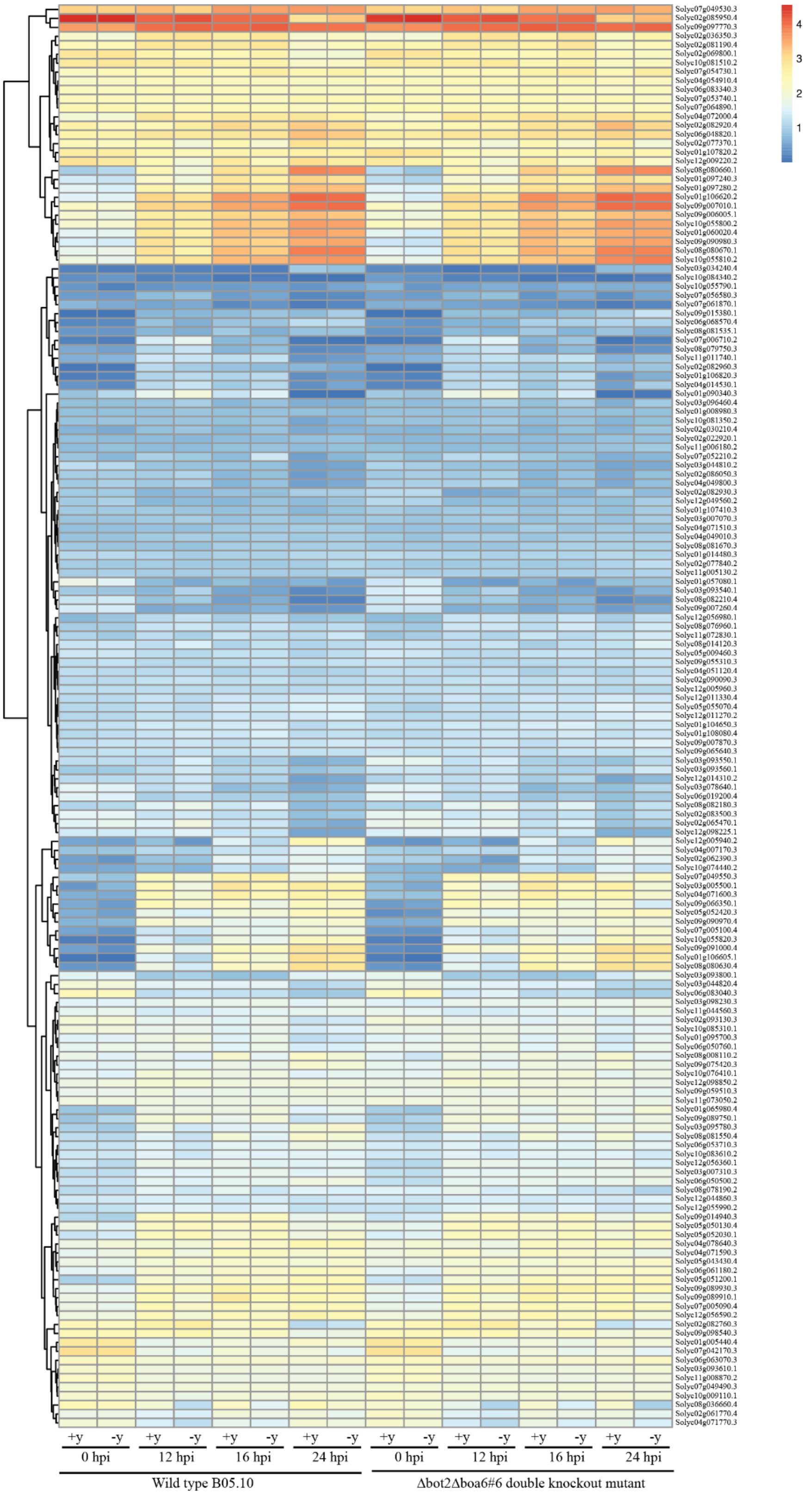


Heatmap of transcript levels of a selected set of 160 tomato genes involved in phytohormone synthesis, perception or signaling, as well as pathogenesis-related protein encoded genes. Corresponding gene list and transcript levels are in Data S2.

**Supplementary Table S1.** Overview of all samples in this study that were used for sequencing

| Sample_name | Strain | Inoculation medium | *In planta* (# of replications) | | | | *In vitro* (# of replications) | | | |
|---|---|---|---|---|---|---|---|---|---|---|
| | | | 0 hpi | 12 hpi | 16 hpi | 24 hpi | 0 hpi | 12 hpi | 16 hpi | 24 hpi |
| WT-y | B05.10 | - y[1] | 3 | 3 | 3 | 3 | 2 | 2 | 2 | 2 |
| WT+y | B05.10 | + y[2] | 3 | 3 | 3 | 3 | 2 | 2 | 2 | 2 |
| ΔΔ-y | *Δbot2Δboa6#6* | - y | 3 | 3 | 3 | 3 | 3 | 2 | 2 | 2 |
| ΔΔ+y | *Δbot2Δboa6#6* | + y | 3 | 3 | 3 | 3 | 3 | 2 | 2 | 2 |

[1] Gamborg B5, 25 mM glucose, 10 mM potassium phosphate, pH 6.0; [2]Gamborg B5, 25 mM glucose, 10 mM potassium phosphate, 0.1 % yeast extract, pH 6.0

**Supplementary Table S2.** Primers used in this study

| Primer name | Sequence (5'-3') | Description |
|---|---|---|
| BcNiaDsgRNA1 | AAGCTAATACGACTCACTATAGGAGGAAAACGATATTCGACGAGTTTTAGAGCTAGAAATAGCAAG | To synthesize sgRNAs targeting BcniaD, both sgRNA1&3 were used to generate transformants to overexpress Bcssp2 in BcniaD |
| BcNiaDsgRNA3 | AAGCTAATACGACTCACTATAGGATGGATGGCCATGAATTCGTGTTTTAGAGCTAGAAATAGCAAG | |
| sgRNA_BcNiiA1 | AAGCTAATACGACTCACTATAGGATCTGCCATCTCGATCCCAGGTTTTAGAGCTAGAAATAGCAAG | To synthesize sgRNAs targeting at BcniiA, both sgRNA1&2 were used to generate transformants to overexpress Bcssp2 in BcniaD |
| sgRNA_BcNiiA2 | AAGCTAATACGACTCACTATAGGTAAAAATCTACCGATCAGGGGTTTTAGAGCTAGAAATAGCAAG | |
| Bcssp2_NcoI_F | TCACAATCGATCCAAccATGGTCCGCATCTCCTCCATCGTC | To amplify Bcssp2 gene with overhangs that can be cloned in pNDH-OGG through NcoI (ccATGG)-NotI (gcggccgc) sites |
| Bcssp2_NotI_R | ATCTACATACGCTAagcggccgcTTACTGCTTGTAACATGGGGTACGAT | |
| Nep2_NcoI_F | TCACAATCGATCCAAccATGGTTGCCTTCTCAAAATCATTACAGCT | To amplify Bcnep2 gene with overhangs that can be cloned in pNAN-OGG through NcoI (ccATGG)-NotI (gcggccgc) sites |
| Nep2_NotI_R | ATCTACATACGCTAagcggccgcCTAGAAAGTAGCCTTCGCAAGATTGTCTG | |
| Bcpg1_NcoI_F | TCACAATCGATCCAAccATGGTTCAACTTCTCTCAATGGCCTC | To amplify Bcpg1 with overhangs that can be cloned in pNAN-OGG through NcoI-NotI sites.<br>Bcpg1_NotI_R was also used with pNDH-PoliC-Fw to check if cloning of Bcpg1 into pNAN-OGG was successful |
| Bcpg1_NotI_R | ATCTACATACGCTAagcggccgcTTAACACTTGACACCAGATGGGAGACC | |
| Xyn11A_NcoI_F | TCACAATCGATCCAAccATGGTTTCTGCATCTTCCCTCCTC | To amplify Bcxyn11A gene with overhangs that can be cloned in pNAN-OGG vector through NcoI (ccATGG) - NotI (gcggccgc) sites |
| Xyn11A_NotI_R | ATCTACATACGCTAagcggccgcTTAAGAAACAGTGATGGAAGCGGAACCA | |
| pNDH-PoliC-Fw | GCTGTGGAGCCGCATTCCCG | Primer in oliC promoter, used with other primers to check if cloning of target gene in the pNDH-OGG/pNAN-OGG vectors was successful |
| pNDH-ssp2-Rv | AGCTGTGAAAGCACTCGCGC | Used with primer pNDH-PoliC-Fw to check if cloning of Bcssp2 in pNDH-OGG was successful |
| Nep2_RT_rev | GGCTCGTCCTTTGGCATGTAC | Used with primer pNDH-PoliC-Fw to check if the cloning of Bcnep2 into pNAN-OGG was successful |

| | | |
|---|---|---|
| BcXyn11A_rv | CGTACGCTTGCTAGTACGGAC | Used with primer pNDH-PoliC-Fw to check if cloning of Bcxyn11A in pNAN-OGG was successful |
| OE-dt-pNDH-3'NiaD | GCAGTTCAATGGCAAAAACTACAAC | To amplify donor template for overexpressing Bcssp2 from re-constructed pNDH-OGG plasmid containing Bcssp2 |
| OE-dt-pNDH-5'NiaD | CATATCAATTGTGTCTCGAGATCGGAAA | |
| pNAN_bcniiA5'_FW | TCATTGAGGCTCTGGTGAACAGTTCA | To amplify donor template for expressing Bcnep2, Bcxyn11A or Bcpg1 from re-constructed pNAN-OGG plasmid containing Bcnep2/ Bcxyn11A/ Bcpg1 |
| pNAN_bcniiA-3'_RV | CGCTAGTATCAATGAGATCATAGAGCTAATGC | |
| pNDH-ssp2-Rv | AGCTGTGAAAGCACTCGCGC | To screen integration of donor template at NiaD site in ΔΔ-OEssp2 transformants. Primer pNDH-ssp2-Rv is in the donor template, NiaD_3'_screenRv is outside flanks of donor template. |
| NiaD_3'_screenRv | GATACTATATTCAAACATCCTCTCTCCC | |
| BcniaD_WTsgRNA1 | ACCTTCGTCGAATATCGTTTTCCTCT | To check if ΔΔ-OEssp2 transformants are homo-karyons, and if OEssp2 donor template is inserted at sgRNA1 cleavage site |
| BcniaD_midRv | GCTAGGAGCTGCGGTAACAA | |
| BcniaD_midFw | CGGAAACCGCCGTAAAGAAC | To check if ΔΔ-OEssp2 transformants are homo-karyons, and if OEssp2 donor template is inserted at sgRNA3cleavage site |
| BcniaD_WT_sgRNA3 | GATGGCCATGAATTCGTCGGTG | |
| Bcniia_screenFw | CTGCTAGACAGATTGTGGCTGGT | To screen integration of donor template at NiiA site in the ΔΔ-OEpg1/nep2/xyn11A transformants. Primer Bcpg1/ Nep2/Xyn11A_NcoI_F is inside of the corresponding donor template, Bcniia_screenFw is outside of flanks of the donor template. |
| Bcpg1_NcoI_F | TCACAATCGATCCAAccATGGTTCAACTTCTCTCAATGGCCTC | |
| Nep2_NcoI_F | TCACAATCGATCCAAccATGGTTGCCTTCTCAAAATCATTACAGCT | |
| Xyn11A_NcoI_F | TCACAATCGATCCAAccATGGTTTCTGCATCTTCCCTCCTC | |
| Bcniia_WT-Fw | GGAATTAAATGCGCTGAACGCG | To check if ΔΔ-Bcnep2/Bcxyn11A/Bcpg1 transformants are homo-karyons in which WT BcniiA is no longer present, primers are inside of flanks of the donor template |
| Bcniia_WT-Rv | GTCGAAGAAACTCCCCACTAGCTC | |

**Legend supplementary data files**

**Supplementary Data S1 (Excel)**
Genes encoding secreted proteins in three modules are positively correlated to the compatible interaction.
Sheet 1 (named “Summary”) contains an overview of the total number of genes encoding secreted proteins in each module resulting from the WGCNA analysis.
Sheets 2-4 contains gene lists for module “lightpink4”, “bisque4”, and “ivory”, respectively.

**Supplementary Data S2 (Excel).** Transcript levels of 160 tomato genes involved in defense responses, phytohormone production, signalling and response, as well as pathogenesis-related proteins. Transcript levels are provided as TPM (average of three biological replicates per sample). Fold changes are provided for comparison between WT and *Δbot2Δboa6#6 B.* cinerea, as well as between inoculation in medium without or with yeast extract. Levels that are two-fold different between treatments are highlighted.
The list of 160 tomato genes that was analysed is provided in the second sheet (Solyc gene IDs and description).
